# Qualitative and Quantitative Analysis of Polyphenols in *Lamiaceae* Plants—A Review

**DOI:** 10.3390/plants7020025

**Published:** 2018-03-26

**Authors:** Katerina Tzima, Nigel P. Brunton, Dilip K. Rai

**Affiliations:** 1 Department of Food BioSciences, Teagasc Food Research Centre Ashtown, D15 KN3K Dublin, Ireland; Aikaterini.Tzima@teagasc.ie; 2 UCD Institute of Food and Health, University College Dublin, Belfield, Dublin D04V1W8, Ireland; nigel.brunton@ucd.ie

**Keywords:** *Lamiaceae*, herbs, polyphenols, liquid chromatography, mass spectrometry

## Abstract

*Lamiaceae* species are promising potential sources of natural antioxidants, owing to their high polyphenol content. In addition, increasing scientific and epidemiological evidence have associated consumption of foods rich in polyphenols with health benefits such as decreased risk of cardiovascular diseases mediated through anti-inflammatory effects. The complex and diverse nature of polyphenols and the huge variation in their levels in commonly consumed herbs make their analysis challenging. Innovative robust analytical tools are constantly developing to meet these challenges. In this review, we present advances in the state of the art for the identification and quantification of polyphenols in *Lamiaceae* species. Novel chromatographic techniques that have been employed in the past decades are discussed, ranging from ultra-high-pressure liquid chromatography to hyphenated spectroscopic methods, whereas performance characteristics such as selectivity and specificity are also summarized.

## 1. Introduction

Concerns over possible adverse health effects of commonly used synthetic antioxidants such as butylated hydroxytoluene (BHT) or butylated hydroxyanisole (BHA) have driven research interests towards finding antioxidants from natural sources, mainly from commonly consumed foods [1,2]. Terrestrial plants constitute one of the most valuable sources of natural antioxidants in addition to other health-promoting phytochemicals [3]. In particular, herbs and spices have shown strong antioxidant activities owing to their high content of polyphenols [2,4].

Considerable attention has been paid to the bioactive compounds in herbs and spices in an effort to reveal their potential contribution to health and the preservation of food quality [5,6]. Several previous studies have suggested that polyphenols from natural sources could be a potential alternative to the use of synthetic antioxidants [3,4]. These antioxidants have many advantages over their synthetic equivalents including consumer acceptance, and the reduced regulatory requirements based on their safety [7]. Natural antioxidants from various botanical sources have been regularly reviewed by focussing on a single species, genus, origin, popularity, applications, bioactivities, selected phytochemical groups of antioxidants, etc. [2]. For instance, *Lamiaceae*, one of the largest herbal families worldwide (236 genera and approximately 6900–7200 species) [8], has been the subject of numerous studies that demonstrated the high radical scavenging capacity (RSC) of its extracts. 

Over the last decade, great effort has been devoted to the development of functional food products that can confer positive health-benefits over and above basic nutrition to consumers [9]. Epidemiological findings as well as scientific data have shown that a diet rich in polyphenols, such as flavonoids and hydroxycinnamic acids, has effective health effects [10,11,12,13] and could confer protection against the risks of degenerative diseases, e.g., cardiovascular diseases [12]. Therefore, further studies are essential in streamlining the various stages of novel functional food formulations, through improving their health benefits and assuring antioxidant and antimicrobial safety [3,14].

**Figure 1:**
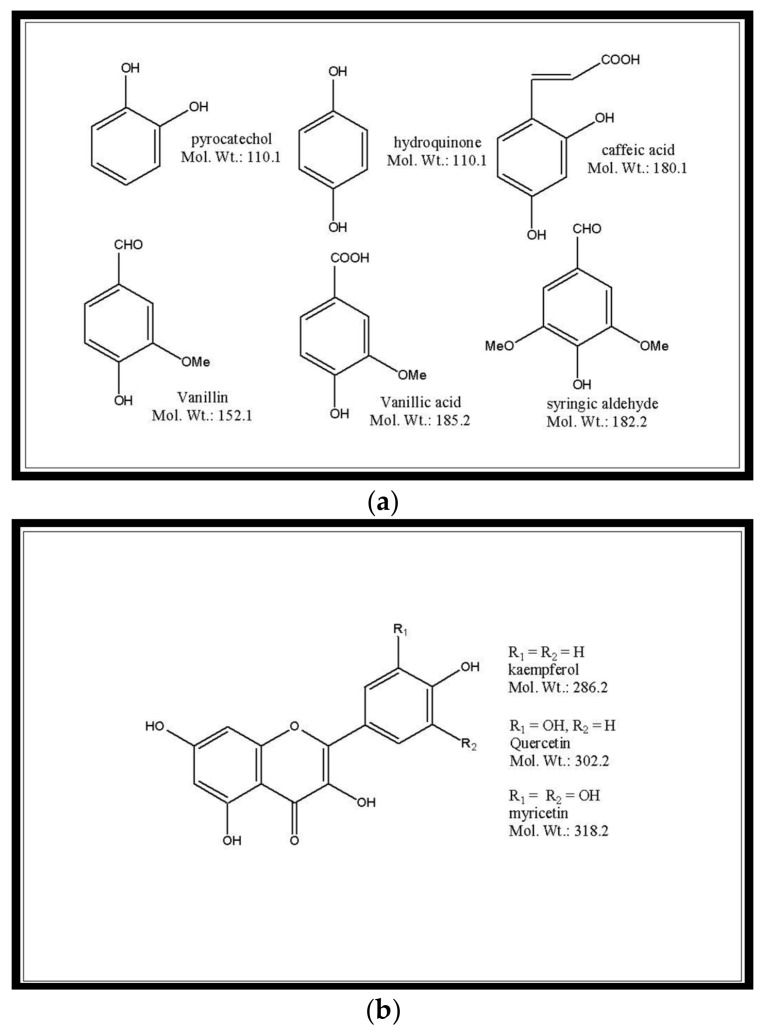

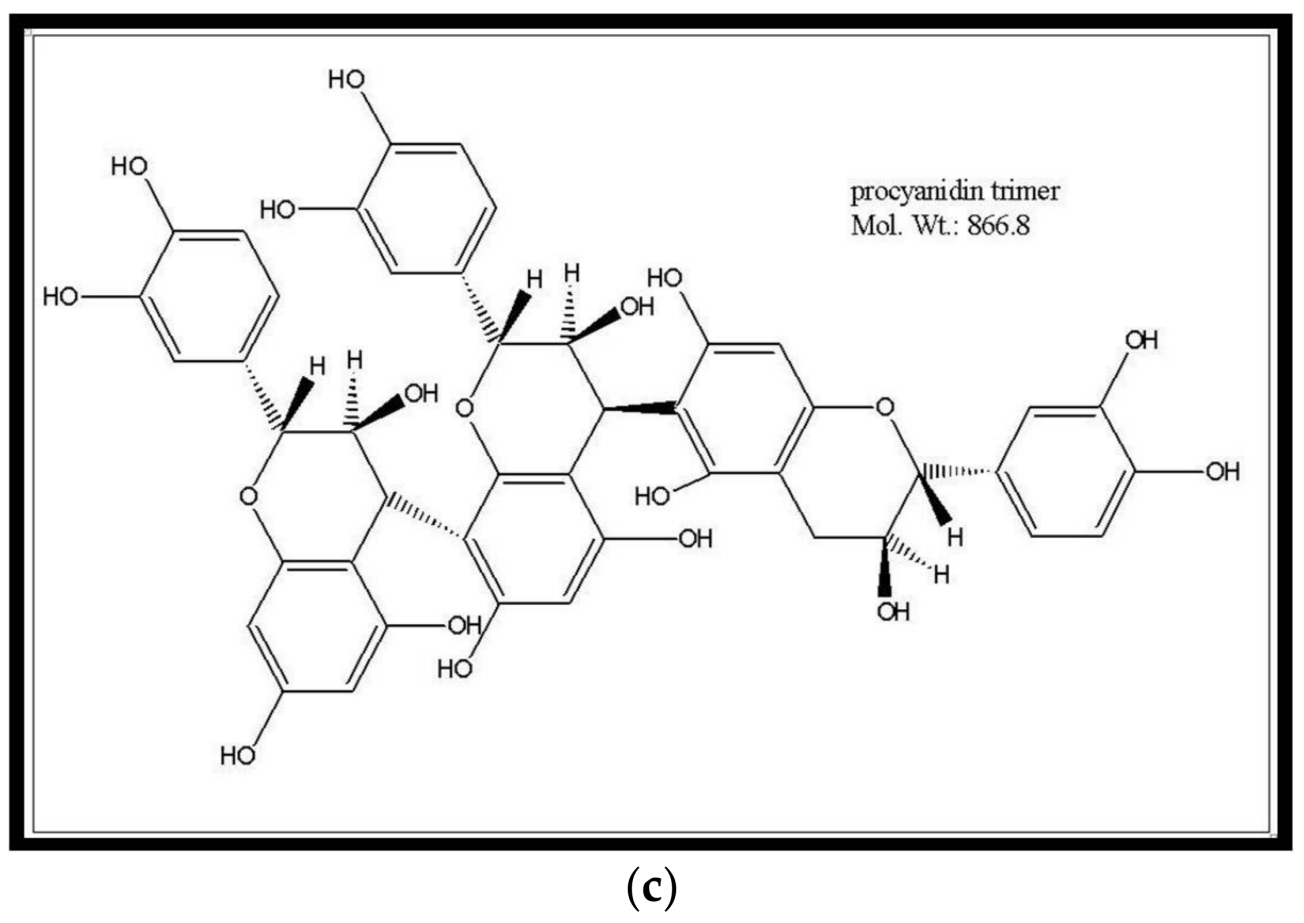
Examples of (**a**) simple and (**b**,**c**) complex polyphenols in plants.

Polyphenols are a group of small organic molecules synthesised by plants as secondary metabolites [15]. These molecules protect the plants from stresses, such as ultra-violet (UV) radiation, infections, cuts, etc. There are many definitions of polyphenols, but the most widely accepted is that “Compounds exclusively derived from the shikimate/phenylpropanoid and/or the polyketide pathway, featuring more than one phenolic unit and deprived of nitrogen-based functions” [15]. Based on this definition, many compounds commonly referred to as polyphenols would not qualify as polyphenols. For example, quinic acid generally listed with polyphenols, is biosynthesized independent of the shikimate pathway Therefore, it cannot be considered as phenolic acid [16]. In the present review compounds such as those presented in Figure 1 will be referred to as polyphenols. Flavonoids, a subset of polyphenols, are characterized by at least two phenol subunits (Figure 1b). The reactive nature of the polyphenols often leads to conjugation with glucose, cellulose, proteins, and with same or other polyphenols forming oligomers (Figure 1c). Several thousand polyphenols have been reported in higher plants [15] and this structural diversity is one of the factors contributing to the complexity of their analysis [17]. Compounded to this is the huge variation in the levels of these compounds in different plant species [3]. The need for sensitive and accurate methods for the analysis of polyphenols is essential, as knowledge of dosage are prerequisites in evaluating health claims of food components.

Classical techniques such as high-performance liquid chromatography (HPLC), thin layer chromatography (TLC), gas chromatography (GC), and capillary electrophoresis (CE), which rely on UV spectrophotometry as the detection tool, have been used for the analysis of polyphenol profiles in herbs [18]. These methods generally lack specificity and sensitivity and rely on the chemical nature of the analytes (chromophore). A common issue being the interference by plant/biological matrices in the UV-dependent assays such as TLC, CE, and HPLC. This has led to an interest in mass spectrometry (MS) coupled with either liquid chromatography (LC) or GC, which has the added advantages of specificity and sensitivity [19]. This review describes the recent (2013–2018) developments and applications of analytical methods in qualitative and quantitative studies of polyphenols following extraction, with special focus on the *Lamiaceae* spices.

## 2. Extraction and Purification

The choice and collection of plant tissues constitute the initial steps for the identification and quantification of bioactive compounds [20]. In order for an analytical technique to generate sufficient data for the determination of natural substances such as polyphenols in plants, it must be sufficiently efficient, selective and sensitive [21]. In this regard, sample preparation is a crucial step before analysis [22], while the sensitivity of the analytical technique is dependent on the polyphenol extraction choice, the purification steps, and the initial concentration of polyphenols in the plant crude extracts prior to analysis [23]. Ideally extraction should result in the selective separation of the target components with high recovery and reduced interferences [24]. Extracts can be obtained with several solvents [20], either organic or inorganic, which can determine the quantity of the extracted phenolics [25]. The most crucial aspect that should be considered for the solvent choice is the polarity of the targeted compounds [26]. Nonetheless, various other factors such as extraction time, temperature, extraction steps, solvent-to-sample ratio [25], molecular affinity among solute and solvent, and use of co-solvents [26] may additionally influence the extractability of phenolics [25]. The optimal content of phenolics is also dependent on the nature of the plant matrix and its bioactive constituents [25]. Plant bioactives can be recovered with several conventional extraction methods, including maceration, distillation, Soxhlet extraction [26], reflux extraction [27], and low pressure solvent extraction (LPSE) [28]. However, these techniques are labor-intensive as they require extended extraction times, large quantities of solvents, and they commonly result in low extraction yields and reduced selectivity [29,30,31,32]. In parallel, the extracts may be subjected to excessive oxygen (O_2_), heat and light, leading to their subsequent degradation [27,29]. Regardless of their inherent multiple drawbacks, liquid-liquid and solid-liquid extraction procedures are still regularly employed [33].

Several novel extraction methods have been established for the recovery of phenolics from plant materials, including microwave-assisted extraction (MAE), supercritical fluid extraction (SFE) [32,34], ultrasound-assisted extraction (UAE) [34] and accelerated solvent extraction (ASE) [29]. In recent years, the use of MAE has gained considerable popularity due to its benefits of diminution of extraction time, reduced cost, sustainability, as well as potential for automation or on-line connection to analytical instrumentation [34,35,36]. Nonetheless, there are certain drawbacks regarding its use in the recovery of polyphenols, in particular the various parameters that could potentially affect its effectiveness, such as the microwave utilization time and power, surface area of the sample, temperature, nature of sample matrix and sample purity [37]. UAE constitutes one of the most simple and convenient extraction processes employing mechanic vibrations generated by sound waves (>20 kHz) for extracting bioactive compounds [25,32]. Nevertheless, in some cases it has been reported that a prolonged sonication (>40 min) in frequencies above 20 kHz could have a detrimental effect on the targeted components. This effect was ascribed to the reduction of diffusion area and rate, but also the increased diffusion distance, which may lead to minimized yield of total phenolics and flavonoids. Furthermore, a potential formation of free radicals may occur [38]. For ASE extraction techniques, low-boiling solvent/solvent mixtures in parallel to increased temperature (>200 °C) and pressure (3000 psi/206.8 bar) are employed. This results reduced solvent viscosity and tension with a parallel elevation of the solvent diffusion rate, mass transfer, and solubility of the targeted components are accomplished. Compared to conventional extraction techniques, ASE utilizes reduced solvent quantities, is time-efficient and automated, and protects the samples from exposure in O_2_ and light [29]. The different characteristics of the SFE extraction process, including the utilization of low temperatures, the absence of O_2_, and the common use of carbon dioxide (CO_2_) render it as a superior procedure for extracting bioactive components [39]. As CO_2_ is economic, non-toxic, nonflammable, and volatile, it may be used in various conditions [40]. In the case of volatile compounds in plant materials such as phenolic terpenes, an extraction process that can be employed is purge and trap (P & T) [41]. This dynamic technique is dependent on bubbling through the sample by using an inert gas such as helium or nitrogen (N_2_). Subsequently, the volatile components of the sample are adsorbed on a trap that is directly heated to desorb them into a gas chromatograph injector [42]. The P & T technique is efficient and results in increased extractability [41].

Matrix effects (ME) constitute a significant disadvantage of LC-MS analysis that the matrix can cause suppression or enhancement of ionization, and subsequent quantification errors [43]. Purifications steps are used to eliminate matrix interferences such as lipids, carbohydrates, or undesirable molecules, and involve the removal of interfering components from the crude extract with an adsorption-desorption process or partitionable solvents (chloroform, hexane, dichloromethane) and open column chromatography [19,44]. Amberlite resin and solid phase extraction (SPE) cartridges are also frequently used materials for the purification of phenolics from crude extracts [44]. With the use of SPE, several disadvantages related to liquid-liquid extraction including use of excessive quantities of solvents, incomplete phase separations, and poor recoveries can be overcome [25]. Although, SPE is commonly employed for the removal of non-phenolic compounds such as sugars, organic acids, and other water-soluble components, this will also lead to the loss of highly polar phenolics [11,44,45]. In addition, there are also costs involved on the SPE manifold and the associated consumables [25]. Table 1 summarizes the extraction processes that were found in the recent literature, regarding the extraction of (poly) phenolic compounds from *Lamiaceae* herbs prior to chromatographic analysis.

## 3. Chromatographic Techniques with Ultraviolet/Visible (UV/Vis) Based Detection

Chromatography, in particular HPLC, is still the most widely used analytical tool for the identification and quantification of polyphenols, which are inherently chromophoric in nature [17,71,72]. In LC, some characteristics of eluted polyphenols can be archived using the detection system, depending on the chemical structure of the molecule. For example, UV/Vis absorption spectra in parallel to the retention time can, with the use of authenticated standards, contribute to the identification of polyphenols in *Lamiaceae* herbs [72].

The separation of phenolics has been improved with the use of reversed-phase (RP) columns (mainly RP C18); however C8 and C12 columns have also been investigated in herbal analysis [73,74,75]. Typical C18 columns in most of the reported HPLC analysis are 100–200 mm length, internal diameters of 3.9–4.6 mm, and stationary phase particle sizes equal to 3–10 µm [23]. A summary of recently reported researches employing conventional as well as hyphenated chromatographic techniques for the qualitative and quantitative analysis of (poly) phenolic compounds in *Lamiaceae* herbs is presented in Table 2.

Regarding the eluents, organic solvents such as MeOH or MeCN in conjunction with aqueous solvents are used [19]. The use of a H_2_O/MeCN binary rather than H_2_O and MeOH did not show any significant improvement in resolution on the HPLC separation of phenolic acids of methanolic extracts of lemon balm (*Melissa officinalis*) (Table 2). Thus, a combination of H_2_O and MeOH could be used to eliminate the cost and toxicity restrictions of MeCN [49]. Elimination of peak tailing in phenolic profile analysis is achieved through the use of various buffers [19] for eluent acidification, as for instance TFA [49], acetic, formic or phosphoric acids, with concentrations ranging from 0.01% to 6% to be the most frequently reported [19]. In addition to the choice of columns and solvents, a significant parameter that influences the separation of phenolic compounds in chromatography is the column temperature [73]. High temperatures lead to reduced eluent viscosity, resulting in shorter elution times, and thus decreasing the organic solvent consumption [17]. As it has been revealed, a temperature of 30 °C gave rise to improved chromatographic resolution of phenolic acids in *Melissa officinalis* (Table 2), compared to 20 °C and 25 °C [49]. Nonetheless, the maximum column functional temperature is 60 °C, whereas higher temperatures could significantly decrease the estimated column life time [69] and may lead to thermal degradation of targeted polyphenols. Therefore, a column temperature equal to 55 °C was used in the research of Zabot et al. [69] to identify phenolic terpenes in different herbs (Table 2). This study had shown that elevating temperature led to a proportional mean reduction of the retention times of the analytes, and accordingly to lower peak widths, increased peak height and an enhanced chromatographic resolution [69]. 

Many studies had been published in the past concerning the elucidation of phenolic profiles of various *Lamiaceae* herbs and spices through HPLC or RP-HPLC [76,77,78,79,80,81]. Nonetheless, more recent studies have also employed these techniques for the same purpose. HPLC analysis with a UV-diode array detector (DAD) was used by Chan, Gan, and Corke [13] for the examination of free (unbound) and bound phenolics (Table 2) in extracts of wild marjoram or oregano (*Origanum vulgare*) and additional herbs and spices [13], considering that bound phenolics encompass a considerable amount of the total phenolics in a matrix [82]. RP-HPLC coupled to UV/Vis-DAD was employed in the research of Žugić et al. [46] and elucidated 12 phenolic compounds in various plants, including European pennyroyal mint (*Mentha pulegium*) and hairless cat-mint (*Nepeta nuda*) (Table 2) [46]. Recently, Skendi, Irakli, and Chatzopoulou [50] developed a simple and reliable RP-HPLC technique with satisfactory sensitivity, reproducibility, accuracy and precision (Table 2) for the qualification and quantification of 24 phenolic compounds in botanicals of the *Lamiaceae* family, by optimizing the mobile phase composition and improving the separation of chromatographic peaks. The limit of detection (LOD) and limit of quantification (LOQ) were sufficiently low for identifying and qualifying low quantities of phenolic compounds, whereas the linearity was also good (*R*^2^ ≥ 0.9961). The phenolic content of the methanolic and aqueous extracts of the studied species declined as follows: Greek oregano (*Origanum vulgare* ssp. *hirtum*) > conehead thyme (*Thymus capitatus*) > winter savory (*Satureja thymbra*) > *Melissa officinalis* > rosemary (*Rosmarinus officinalis*) [50]. An HPLC method with UV/Vis detector was also developed and validated by Arceusz and Wesolowski [49] to evaluate the quality consistency of *Melissa officinalis*. Commercial herbs, while the optimized HPLC method was employed for the separation, identification and quantitation of six phenolic acids detected in this herb (Table 2) [49].

In the recent years, on-line HPLC-2,2, diphenyl-1-picrylhydrazyl radical (DPPH•) assay had been additionally used to effectively screen for the fast identification of antioxidant compounds from herbal extracts [83,84]. A simultaneous detection and quantification of compounds in complex plant matrices with high antioxidant potentials have also been investigated through on-line HPLC-UV-DPPH• analysis [2,3]. This technique was used by Damašius et al. [2] on extracts from different species of *Lamiaceae* family (Table 2). The authors concluded, that a strong correlation was found between antioxidant levels using the DPPH• bulk assay with that measured by the summed peak area attained through the on-line HPLC/UV/DPPH•. One phenolic acid, i.e., lithospermic acid B, was identified for the first time in marjoram (*Origanum majorana*), savory (*Satureja hortensis*) and thyme (*Thymus vulgaris*) (Table S1) [2]. The same technique was used adapted by Šulniūtė, Pukalskas, and Venskutonis [3] to identify rapidly the compounds with antioxidant potential in the extracts of different sage species (*Salvia* spp.) [3].

With advances in chromatography technologies in the past decade, ultra-high performance liquid chromatography (UHPLC) has enabled rapid separation of phenolics with much reduced time and cost [52]. UHPLC or UPLC is a chromatographic technique that is commercially available since 2004, and its applications have been rising steadily also for the qualification and quantification of the major phenolic compounds of several *Lamiaceae* herbs and spices [85,86,87]. The capability of higher pressure that ranges up to 15,000 psi (1035 bar) [86,88] and smaller particle size (potentially lower than 2 μm) [55,86,88], result in more rapid [55,86,88,89] effective [86], and sensitive separation of analytes [88]. Besides HPLC and UHPLC, there are other chromatography-based separation techniques that have been employed for phenolic profile characterization, such as CE and TLC. These techniques, in particular CE, can also be hyphenated to MS for acquisition of structural data [72]. 

TLC is a rapid and easy-to-use technique that can be employed for initial identification of phenolics in various extracts [82]. Even if the popularity of TLC has decreased as a result of the advance of column chromatography, it remains an essential tool in the research of polyphenols in natural extracts [58]. TLC and HPLC with DAD detection system were used by Fatiha et al. [57] in order to diminish the probability of misidentification, throughout elucidation of the phenolic profiles of extracts of mint subspecies (*Mentha* spp.) (Table 2). TLC and HPLC analysis revealed similar phenolic compounds (caffeic acid, rosmarinic acids, and diosmin) as well as their derivatives were identified with both techniques in all extracts [57]. Jesionek, Majer-Dziedzic, and Choma [58] optimized a TLC technique and separated 10 typical phenolic constituents from five plant species extracts, including *Thymus vulgaris* and common sage (*Salvia officinalis*) (Table 2) [58]. In parallel, a TLC-DPPH• assay was used to define the antioxidant capacity of the extracts, and liquid chromatography coupled to mass spectrometry (LC-MS) as a confirmation tool of the occurrence of the targeted phenolics. The separation of polyphenols on TLC is typically accomplished with silica gel and AcOEt:acetic acid:formic acid:water (100:11:11:26, *v*/*v*) as a mobile phase. Nonetheless, seven different mobile phases were used to optimize the separation of polyphenols, while two novel were ultimately established and utilized. The optimized eluent system enabled the good separation of phenolic compounds and correspondingly their clear detection. Apigenin 7-*O*-glucoside was the only phenolic compound that did not display any antioxidant capacity through TLC–DPPH• assay, while most likely, the low concentration of the four additional phenolic constituents identified through LC-MS was the factor that restricted their detection through TLC [58].

Regardless of its low resolution [82], TLC represents a valuable technique as it can be easily setup for 2-D chromatography, whereas post-separation derivatization process can deliver further analyte selectivity [72]. Two-dimensional (2D) LC or LCxLC offers enhanced resolution of complex matrices and is becoming extensively utilized due to the improved characterization of compounds with respect to one-dimensional liquid chromatography [90]. In some cases, analysis of phenolic in herbs and spices by conventional chromatographic techniques is challenging especially when key components cannot be effectively resolved, indicating the demand of effective multi-dimensional separation techniques. An LC × LC system is constituted in most of the cases by two different separation columns which results in the efficient qualification and quantification of compounds. Subsequently, improved MS analysis can be achieved as the matrix-associated ionization suppression is minimized [91]. In the research of Hawrył et al. [48], a micro-2D-TLC method with cyanopropyl layers led to the separation of phenolic fractions from several mint species (*Mentha* sp.) extracts (Table 2). The 2D-TLC data indicated the presence of rutin, narirutin, rosmarinic acid, isorhoifolin, diosmin, and naringenin in all the *Mentha* sp. extracts. Initially, the technique was optimized through the utilization of different concentrations of MeCN and H_2_O. Subsequently, the eluents with the higher selectivity were used to optimize the 2D systems through the development of R_f_ (Retention factors) on the TLC plates, for both normal and reversed phases. It was noted that the 2D-TLC technique was highly sensitive, time efficient, and required low volumes of eluent and sample [48].

Separation and analysis of polyphenols in herbs and spices by CE involves separation based on the electrophoretic mobilities of a solution that consists of electrically charged species, in small-diameter capillaries [92] it is recognized as being effective in phenolic characterization, offering practical operation, rapid analysis, low consumption of solvent, and low cost. This method represents a valuable alternative to HPLC in the separation of closely associated phenolics, but its major drawbacks are its lower reproducibility and sensitivity as compared to HPLC [93]. Maher et al. [47] used an optimized CE with DAD to identify luteolin and apigenin in *Thymus vulgaris* and an additional herb extract (Table 2). The technique was optimized in terms of voltage, capillary temperature, applied pressure, detection wavelength, as well as pH and buffer, and MeOH concentration. The principal advantages of the CE technique were its selectivity for the analytes, deprived from interferences from other compounds, its short analysis time (less than 35 min) and the ease of use. In parallel, it was characterized as sensitive, accurate and precise [47].

## 4. Hyphenated Chromatographic Techniques

Over the last two decades, hyphenation of chromatographic and spectroscopic techniques has gained considerable esteem in the analysis of complex biological matrices [94]. Mass spectrometer coupled to LC or GC constitutes the most widely used hyphenated analytical methods in the analysis of food components [95]. The basic principle of MS is the generation of ions in gas phase from either organic or inorganic compounds, the separation of ions based on their mass-to-charge ratio (m/z) and the qualitative and quantitative detection of the components through their respective m/z and abundance [96]. For the molecules that do not ionise readily, atmospheric pressure chemical ionization (APCI) to assist ionization has been used in the LC-MS methods [65,97,98,99].

LC-MS [3,100,101,102] and LC-MS/MS [103,104,105] have been widely used for the characterization of the phenolic profiles of various herbs and spices. LC-DAD-MS was used by Atwi et al. [66] to analyse three sage (*Salvia*) species (Table 2), native in Crete Island (Greece), in AcOEt and *n*-butanol extracts. As the chromatographic analysis revealed, the different species had a high phenolic content, predominantly in flavones, while a restricted amount of phenylpropanoids was also present. Additionally, Greek sage (*Salvia fruticosa*) *n*-butanol extracts showed the highest antioxidant capacity [66]. In addition, Milevskaya et al. [70] used LC-DAD-MS analysis to qualify and identify the extracted phenolic compounds from 4 *Lamiaceae* herbs, namely *Salvia officinalis* L., creeping thyme (*Thymus serpyllum*), *Origanum vulgare*, and *Melissa officinalis*) by utilizing different extraction processes (Table 2). Subcritical extraction resulted in the highest extractability of phenolics, while *Origanum vulgare* exhibited the maximum content in some of them. Nonetheless, the researchers also suggested that the comparison of the UV spectra and retention times of analytes and standards is not adequate for qualifying phenolics in medicinal plants, while the supplementary use of MS could provide higher reliability to the process [70]. Tuttolomondo et al. [61] applied HPLC-PDA/ESI-MS on the analysis of phytochemicals in 57 wild Sicilian oregano (*Origanum vulgare* ssp. *hirtum*) samples (Table 2), where 13 polyphenol derivatives (flavanones, flavones, organic acids) were quantified and showed that flavanones were more abundant that the flavones [61]. In the subsequent studies by the same research group on wild Sicilian *Rosmarinus officinalis* L. [62] and wild Sicilian thyme (*Thymus capitatus* L.) [63], eighteen compounds (flavones, diterpenes, organic acids) and fifteen flavonoid derivatives were identified in the respective Lamiaecea species examined [62, 63].

LC-MS/MS was used by Sonmezdag, Kelebek, and Selli [64] for the characterization of the phenolic compounds of *Thymus serpyllum* (Table 2), after aqueous-alcoholic extraction, where 18 phenolic compounds were identified and quantified; of which 10 of the 18 compounds were reported for the first time in this species (Table S1). Except for luteolin 7-*O*-glucoside that was the predominant compound of the phenolic fraction, luteolin and rosmarinic acid were also detected in considerable quantities [64]. In another study, Hossain et al. [60] employed LC-ESI-MS/MS (Table 2) to qualitatively and quantitatively examine antioxidant-guided polyphenol rich fractions of *Origanum majorana*, following flash chromatography (FC). The study revealed that rosmarinic acid, confirmed with ^1^H nuclear magnetic resonance (NMR) data, mainly attributed to the antioxidant activity of *Origanum majorana* [60]. FC constitutes on of the simplest methodologies of maximizing the quantities and purity of natural active isolates, for their supplementary structural interpretation through NMR spectroscopy. Regardless its lower resolution compared to other techniques, FC has the benefits of being simple and inexpensive [106].

NMR spectroscopy is often used as a confirmatory tool in the identification of polyphenols [19]. NMR analysis is essential to establish the configuration of new molecules that have been reported for the first time, by measuring the total biochemical composition of a matrix [18,72]. However, the limiting factor for elucidation of chemical structures through NMR is the requirement of high quantities of the substances with excellent purity [72]. Particularly, ^1^H NMR spectroscopy can deliver rapid, direct and without interferences profiling of polyphenols [82]. A combination of HPLC-DAD ESI-MS, MS^n^ and 2D-NMR (^1^H, ^13^C) analysis [59] were employed in profiling phenolic compounds of lemon thyme (*Thymus* x *citriodorus*) ethanolic extracts (Table 2). The in-house validation of this combined method gave rise to sufficient results of linearity (adjusted *R^2^* values ~0.999), instrumental and technique precision as well as accuracy, whereas LOD and LOQ values revealed an adequate sensitivity for all used phenolic standards. Among the 13 identified phenolics in *Thymus* x *citriodorus*, the major compound was rosmarinic acid. However, luteolin-7-*O*-glucuronide was also detected in high quantities for the first time in thyme (*Thymus*) species (Table S1), whereas other novel compounds were also present (Table S1) [59]. Several studies have demonstrated the application of UHPLC-MS/MS for phenolic profiling of herbal samples [55,64,66,68] which is deliberated as advanced, sensitive, reproducible, rapid and with high resolution technique [68]. For instance, Mena et al. [53] have used UHPLC-ESI-MS^n^ with a total run time of 35 min, for the phenolic compositional analysis of a branded extract of *Rosmarinus officinalis* (Table 2), where 57 compounds were identified and quantified, and of which 14 polyphenols were detected for the first time (Table S1) in this species [53]. In another UHPLC-ESI-MS^n^ study of methanolic extracts of dried *Mentha spicata* L., by Cirlini et al. [56], its (poly) phenolic profile was fully elucidated (Table 2), and 66 molecules were identified, whereas 53 of them were semi-quantified in a shorter time, equal to 20 min [56]. Compared to the conventional LC systems, UHPLC based separation methods are five to 10-fold faster with peak resolutions retained [89] or enhanced [55,88,89] whereas they result in lower limits of detection and reduced solvent consumption [14]. The benefits of these techniques stem from the used analytical columns, with particle size <2.0 μm, which lead to considerable reduction of back-pressure [14]. Polyphenolic profiles of *Lamiaceae* species, namely *Origanum majorana*, *Mentha pulegium* and lavender (*Lavandula officinalis*) were also scrutinised by Çelik et al. [68] on MAE 60% MeOH extracts (Table 2). The authors optimized and validated the UHPLC-DAD-ESI-MS/MS method that had a total run time of 12 min per sample. A total number of 18 polyphenols was identified in the samples and the technique exhibited good reproducibility (recoveries equal to 92–109%) and linearity (r ≥ 0.9988), whereas LOD and LOQ values of polyphenols were diminished to 0.02 ng/mL and 0.06 ng/mL, respectively. The advantages of this method over HPLC are attributed to the reduction of analysis time and its applicability to a greater number of polyphenolic compounds [68]. Oliveira et al. [14] developed and validated an UHPLC-DAD method (Table 2) that enabled for the first time the simultaneous quantification of 19 phenolic compounds in 21 fresh and dried (organic and non-organic) aromatic plants, most of them belonging to *Lamiaceae* family. This technique was capable of identifying and quantifying phenolic compounds at a concentration <0.15 μg/mL, apart from carnosol and carnosic acid, in a relatively short run time (30 min), whereas it was direct, sensitive, with good precision, accuracy and linearity. It was further revealed, among the different aromatic plants, *Thymus vulgaris* displayed the highest range of different phenolics [14].

Even if reduced particle size leads to increased column efficiency and analysis time, it also results in increased back-pressures. Fused-core technology is considered as a way of archiving both the benefits of small particles and the existing pressures with an HPLC system, consisting of 1.7 µm solid silica bead surrounded by a 0.5 µm porous shell, while deriving a particle size equal to 2.7 µm. One benefit of the fused-core columns is that for a certain column length, it does not involve the comparatively high pressures that are essential by a column packed with 1.7 µm material. Nonetheless, the overall column efficiency is reduced by 20% in comparison to a 1.7 µm packed bed [107]. Zabot et al. [69] employed UHPLC-MS to confirm the identified phenolic terpenes, while developing and validating a rapid HPLC-PDA technique through a fused-core column for their analysis in *Rosmarinus officinalis* (Table 2). Several chromatographic parameters were optimized (column temperature, gradient and flow rate, re-equilibration period), and the validated technique had the ability to detect and quantify the principal non-volatile constituents of *Rosmarinus officinalis* (carnosol, rosmanol, carnosic acid, rosmarinic acid, methyl carnosate) in low amounts of 0.25 µg/mL and 1 µg/mL, respectively. The analysis had a short total run time of 10 min and was shown to be convenient in use, selective, robust and reliable [69].

Liquid chromatography coupled to various mass spectrometers such as TOF, and Orbitrap attracting considerable interest the last years [108], rendering high resolution mass spectroscopy (HRMS) as a powerful structural elucidation tool [109]. The contemporary hybrid mass analysers, such as Q-TOFs and Q-Orbitraps, have led to remarkable technological developments in facilitating specific ion fragmentation and expedite data mining and thereby increase the potential for the identification of unknown compounds [110]. Except for providing improved specificity compared to conventional MS techniques, HRMS techniques correspondingly facilitate software expedite data mining. Even if reference standards are essential for conformation of identity, when they are absent, these methods have the capacity to tentatively or fully identify the unknown compounds [55,111] based on UV absorption, MS spectra and information in the literature [55]. LTQ-Orbitrap-MS is the most advanced mass spectrometry technique that allows rapid, accurate and sensitive structural elucidation of small molecules [11,112], without the effect of the relative ion abundance [112] and through MS, MS/MS as well as MS^n^ [11]. SPE followed by LC and coupled with ESI-LTQ-Orbitrap-MS [11] resulted in the identification of 52 polyphenolic compounds in several families of culinary herbs and spices including *Lamiaceae* (Table 2), despite the fact that standards were not employed in the analysis [11]. The principal compounds were also quantified through LC coupled to ESI-QqQ and multiple reaction monitoring (MRM mode with optimized conditions. Moreover, two polyphenols were identified for the first time in the examined *Lamiaceae* herbs (*Rosmarinus officinalis*, *Thymus vulgaris*, and *Origanum vulgare*) (Table S1) [11]. The same conditions were effectively used in the subsequent study of Vallverdú-Queralt et al. [51], for the analysis of the phenolic profile of five additional herbs, including *Origanum majorana* (Table 2), whereas 22 phenolics were identified in its extract [51]. Pandey et al. [54] developed an UHPLC coupled to QqQ_LIT_-MS/MS in MRM mode, to investigate differences in the bioactive components, among them (poly) phenolic compounds, of leaf extracts of six basil (*Ocimum*) species (Table 2). The developed and validated technique was rapid, with a run time of 13 min, whereas it was characterized as sensitive, precise and reliable, according to the international standards. Among all the bioactive constituents and for almost all the examined *Ocimum* species, rosmarinic acid was the predominant phenolic constituent [54]. 

The accurate mass measurement of Q-TOF for precursor and product ions, constitute the factors of its wide application [113]. Extracts of leaves of 20 *Rosmarinus officinalis* plants originated from different areas of Serbia were analyzed by high performance liquid chromatography coupled to HPLC-ESI-Q-TOF-MS and MS/MS (Table 2) by Borrás-Linares et al. [67] Q-TOF mass analyzer resulted in the qualification and quantification of the 30 phenolic compounds (Table 2) and was established as an important detection system in phenolic characterization, offering mass accuracy and true isotopic spectral distribution in both MS and MS/MS [67]. HPLC–ESI–Q-TOF–MS was also employed in the research of López-Cobo et al. [55] and elucidated (Table 2) the phenolic profile of the wild growing winter savory (*Satureja montana* ssp. *kitaibelii*). In this study, a total of 44 phenolics were identified, of which 42 were identified for the first time in this species (Table S1) [55]. Šulniūtė, Pukalskas, and Venskutonis [3] examined 10 *Salvia* spp. species following SFE-CO_2_ in EtOH and H_2_O (Table 2). Subsequent analysis of this extract using UHPLC-Q-TOF and UHPLC-TQ-S was performed and showed that rosmarinic acid was the principal compound in various *Salvia* spp., mainly in ethanolic extracts. Additional polyphenols, i.e., apigenin glucuronide, caffeic and carnosic acids were identified and quantified for the first time in *Salvia* spp. (Table S1) [3]. Methanolic extracts of Tunisian *Mentha pulegium* and *Origanum majorana* were analyzed with UHPLC-Q-TOF-MS by Taamalli et al. [52]. The authors detected 85 metabolites from several chemical families, and among them were phenolic compounds, which were quantified spectrophotometrically based on the chromatographic peak areas. This study had shown higher amounts of polyphenols in *Mentha pulegium* extract than in *Origanum majorana*, and high-resolution mass spectra with accuracy of 5 ppm were delivered. According to the authors, this study enabled the characterization of several compounds belonging to different classes in a single run, and some of the compounds reported for the first time in this species (Table S1) [52]. 

Even if HRMS is effective in the detection of novel compounds, supplementary characterization is required for incontrovertible results, as for instance through the use of ^1^H NMR and ^13^C NMR analysis. Nonetheless, in most of the cases where new compounds are identified, adequate information is available to minimize the selection, attributed to a logical framework for extrapolation from identified compounds to the unidentified [114]. ^1^H NMR, ^13^C NMR including 2D NMR analyses in tandem with LC-MS/MS in MRM acquisition mode were utilized to validate the results of HPLC-PDA and LC-HRMS in the investigation of the phenolic profile of Australian mint (*Mentha australis* R. Br.) (Table 2). MRM mode is particularly specific and more sensitive compared to LC-HRMS. Therefore, it was employed to validate the chemical structures attained through LC-HRMS by scrutinizing the product ions of authentic standards and excluding the unwanted ions. Through this means, it enabled precision while relating to the standards. It was shown in this study that LC-HRMS delivered mass accuracy of less than 2 ppm. Except for rosmarinic acid and neoponcirin, gallic acid equivalent, narirutin, chlorogenic acid, and biochanin A were also identified as major compounds of *Mentha australis* R. Br., whereas two phenolic compounds were identified for the first time in the *Mentha genus* (Table S1) [65].

GC is also used in some cases for the quantification of phenolic compounds, in particular for volatiles [71]. Generally, fused silica capillaries of 30 m length and internal diameters of 25–32 µm, and a stationary phase particle size of 0.25 µm are used in GC. Flame ionization detector (FID) and MS are the commonly used detectors [23]. Although GC has been used particularly for identification and quantification of flavonoids and phenolic acids, the low volatility of phenolics is a deterrent factor requiring chemical derivatization (methylation) [44]. GC coupled to MS has been used in profiling phenolics in herbs and spices [23]. Two phenolic terpenes (thymol and carvacrol) were the main compounds in the essential oil of *Thymus serpyllum* as determined by GC-MS (Table 2). The volatile compounds were recovered, and their separation was carried out using a flame ionization detector (FID) and a mass-selective detector (MSD). Subsequently, the aroma extract dilution analysis of the extract was followed with GS-MS-O [64]. The GC-MS-O technique provides separation of the volatile compounds by odorous and non-odorous properties, based on their concentrations in the examined matrix [64]. In a separate study by Tuttolomondo et al., GC-FID and GC-MS analyses showed 81 compounds in the essential oils of wild Sicilian *Origanum vulgare* ssp. *hirtum*. obtained after hydrodistillation, and the principal compound in the extracted oils was the phenolic terpene thymol [61]. In the following studies by Napoli et al. [62] and Saija et al. [63], GC-FID and GC-MS analyses on wild Sicilian *Rosmarinus officinalis* L. and *Thymus capitatus* L. identified carvacrol as the major phenolic terpene in *Thymus capitatus* L. oils [63].

## 5. Conclusions

The promising results in last decades regarding the antioxidant and health-promoting properties of *Lamiaceae* merit the investigation of their active compounds, which are predominantly polyphenols. Advances in analytical technologies, such as hyphenated methods and multi-dimensional separation techniques, including UHPLC or LC x LC coupled to MS such as Orbitrap and Q-TOF, or NMR, have enabled the identification of several new polyphenols in *Lamiaceae* herbs, and in addition made it possible to quantify the low levels (nanograms) present in some matrices. Nonetheless, further development in analytical capabilities is required to distinguish the structural anomaly diversity of polyphenols and their metabolites (transformed by gut bacteria or enzymes) in a complex matrix.

## Figures and Tables

**Table 1:** Extraction processes for polyphenolic constituents from *Lamiaceae* herbs.

i) *Lamiaceae* Species ii) Plant Part	Extraction Process	Polyphenol Classes	i) Solvent ii) Solute: Solvent Ratio	i) Time (t) ii) Temperature (T)	i) Work-up and Conditions ii) Purification/Clean-up ^1^	Reference
i) *Mentha pulegium;* *Nepeta nuda* ii) Aerial parts	Reflux condensation	Phenolic acids; Flavonoids	i) methanol (MeOH) ii)1:10 weight/volume (*w*/*v*)	i) 30 min ii) not available (n/a)	i) Exhaustive-extraction (two times); Filtration ii) n/a	[46]
i) *Thymus vulgaris* ii) Aerial parts	Reflux (hot) extraction	Flavonoids (flavones)	i) MeOH ii) 1:6 (*w*/*v*)	i) n/a ii) n/a	i) 3 Extraction Repetitions; Drying (rotary evaporator); Reconstitution of residue (1.5 g residue: 5 mL MeOH); Filtration; Dilution (1:2) with 0.5 mL borax buffer (20 mM, pH 10.0) ii) n/a	[47]
i) 11 species of *Mentha*; 2 Mixtures of *Mentha* species ii) Plant material; Pharmaceutical products	Soxhlet extraction of residue after chlorophyll removal	Hydroxycinnamic acids; Flavonoids	i) MeOH ii) 1:10 (*w*/*v*)	i) 8 h ii) n/a	i) Evaporation (water bath, 0.9 atm); Dissolution of residue to 25 mL with MeOH ii) Isolation of Chlorophylls: Soxhlet extraction with chloroform, 8 h, 20 g of solute	[48]
i) *Melissa officinalis* ii) Fresh herbs or leaves	Sonication	Hydroxybenzoic, Hydroxycinnamic acids	i) 80% aqueous MeOH ii) 1:8 (*w*/*v*)	i) 30 min ii) ambient	i) Centrifugation (20,000 rpm, 10 min); Two process repetitions; Combination of extracts; Dilution (Final volume: 25 mL, with 80% aqueous MeOH); Filtration ii) n/a	[49]
i) *Origanum vulgare* ssp. *hirtum*; *Thymus capitatus*; *Satureja thymbra*; *Melissa officinalis*; *Rosmarinus officinalis* ii) Aerial parts, dried, grounded leaves and flowers	Sonication	Phenolic acids and their derivatives; Flavonoids; Phenolic monoterpenes	i) 70% aqueous MeOH or water (H_2_O) ii) 1:8 (*w*/*v*)	i) 20 min ii) ≤30 °C	i) Centrifugation (12,500 rpm, 15 min, 4 °C); Filtration ii) n/a	[50]
i) *Rosmarinus officinalis*; *Origanum vulgare*; *Thymus vulgaris*; *Origanum majorana* ii) Dried, grounded	Sonication	Flavonoids; Phenolic acids; Phenolic terpenes	i) 0.1% formic acid in 50% aqueous ethanol (EtOH) ii) 1:5 (*w*/*v*)	i) 5 min ii) n/a	i) Centrifugation (3000 g, 10 min, 4 °C); Two repetitions (residue); Combination of extracts; Evaporation with N_2_; Reconstitution of extracts to 5 mL with 0.1% aqueous formic acid ii) Solid-Phase Extraction (SPE: Dilution (1 mL extract, 1 mL H_2_O, 34 µL 35% hydrochloric acid (HCl)); Equilibration (1 mL MeOH, 1 mL sodium acetate 50 mmol/L, pH 7); Rinsing (sodium acetate 50 mmol/L, pH 7.5% MeOH); Elution of polyphenols (1800 µL 2% formic acid in MeOH); Evaporation (N_2_); Residue dilution to 250 µL with 1% formic acid in H_2_O); Filtration	[11,51]
i) *Mentha pulegium*; *Origanum majorana* ii) Aerial parts	Sonication	Flavonoids; Hydroxybenzoic, Hydroxycinnamic acids and their derivatives	i) MeOH ii) 1:10 (*w*/*v*)	i) 30 min ii) ambient	i) Centrifugation (3500 rpm, 10 min); four repetitions; Collection of supernatants; Evaporation (reduced pressure, 35 °C); Residue re-constitution to 2 mL with MeOH; Filtration ii) n/a	[52]
i) *Rosmarinus officinalis* ii) Branded extract rich in carnosic acid	Sonication	Flavonoids (mainly flavones); Phenolic terpenes (diterpenoids and derivatives); Phenolic acids	i) 2% formic acid in acetonitrile (MeCN) ii) 1:6.7 volume/volume (*v*/*v*)	i) 10 min ii) n/a	i) Centrifugation (10,480 g, 5 min, Ambient T); Direct injection after centrifugation ii) n/a	[53]
i) 6 *Ocimum* spp. ii) Leaves, dried, grounded	Sonication (53 kHz)	Phenolic acids; Flavonoids; Propenyl phenols; Terpenoids	i) 80% aqueous MeOH ii) 1:10 (*w*/*v*)	i) 30 min ii) ambient	i) Maintenance 24 h (22–24 °C); Filtration; Evaporation (reduced pressure, 40 °C) ii) Sonication of the residue (1 mg) in MeCN (1 mL) Filtration (0.22 µm filter); Dilution to 30 ng/mL (MeCN); Spiking (andrographolide).	[54]
i) *Satureja montana* ssp. *kitaibelii* ii) Aerial parts of wild plant, air-dried, milled	Solid-liquid extraction, Sonication	Hydroxybenzoic, Hydroxycinnamic acids; Phenyl acetic acids; Flavonoids (flavones, flavonols)	i) 60%, 70% and 80% aqueous MeOH, EtOH and acetone ii) 1:10 (*w*/*v*)	i) 10 min ii) n/a	i) Centrifugation (1000 g, 15 min); Removal of supernatant and exhaustive extractions (three repetitions); Evaporation of supernatants; Reconstitution in MeOH: H_2_O 50:50 (*v*/*v*) (1 mL); Filtration ii) n/a	[55]
i) *Mentha spicata* ii) Commercial extract	Solid-liquid extraction, Sonication	Hydroxybenzoic, hydroxycinnamic acids; Flavonoids (flavones, flavonols)	i) 80% aqueous MeOH with 1% formic acid ii) 1:5 (*w*/*v*)	i) 25 min ii) ambient	i) Centrifugation (10,480 g, 5 min, ambient T); Exhaustive extraction (three repetitions: on the same sample) ii) n/a	[56]
i) 3 *Mentha* spp. ii) Dried and powdered leaves	Solid-liquid extraction of defatted residues	Phenolic acids; Flavonoids	i) EtOH ii) 1:40 (*w*/*v*)	i) 24 h ii) ambient	i) Filtration (on cellulose); Concentration (vacuum evaporator, 40 °C) ii) Defatting: Stirring (130 rpm); 25 g of sample in *n*-hexane (600 mL); Ambient T; 3 h	[57]
i) *Origanum vulgare*; *Ocimum basilicum*; *Rosmarinus officinalis*; *Origanum majorana*; *Thymus vulgaris*; *Satureja hortensi*s ii) Commercial, dried, grounded leaves	Shaking, Solid-liquid extraction	Phenolic acids	i) 70% aqueous EtOH ii) 1:10 (*w*/*v*)	i) 2 h ii) ambient	i) Filtration; Vacuum evaporation (40 °C); Freeze-drying; Analysis concentration: 0.1% (*w*/*v*) ii) n/a	[2]
i) *Thymus vulgaris*; *Salvia officinalis* ii) Aerial parts	Maceration (herbal tinctures)	Phenolic acids (hydroxycinnamic acids); Flavonoids (flavonols, flavones)	i) 70% aqueous EtOH ii) n/a	i) 7 days ii) n/a	i) (According to the Polish Pharmacopoeia VI protocol) ii) n/a	[58]
i) *Thymus* x *citriodorus* ii) Mixture of leaves and stems, dried	Maceration of residue (defatted)	Phenolic acid derivatives; Flavonoids (flavones, flavonols, flavanones)	i) 80% aqueous EtOH ii) 1:60 (*w*/*v*)	i) 30 min ii) ambient	i) Filtration; Four re-extractions of residue; Combination of extracts; Lyophilization ii) Defatting: Maceration with *n*-hexane (150 mL); 5 g of sample; 30 min; Ambient (T); three repetitions	[59]
i) *Origanum majorana* ii) Commercially produced, dried, grounded	Solid-liquid extraction	Flavonoids; Phenolic acids	i) 80% MeOH ii) 1:10 (*w*/*v*)	i) 6 h, 16 h ii) 23 °C	i) Filtration; Combination of extracts; Drying (rotary evaporator, 50 °C); Dissolution in H_2_O (16.5 g/500 mL) ii) Liquid-liquid partitioning for flash chromatography (FC): ethyl acetate (AcOEt) (500 mL) in H_2_O (500 mL) with 16.5 g of extract; Dissolution of polar part (14.7 g) in H_2_O (50 mL) and non-polar part (1.7 g) in AcOEt (50 mL)	[60]
i) *Rosmarinus officinalis*, *Origanum majorana*, *Origanum vulgare Ocimum basilicum*, *Mentha spicata*, *Thymus vulgaris Mentha* x *piperita*, *Thymus* x *citriodorus* ii) Fresh; Dried; Organic dried	Solid-liquid extraction aided by shaking	Hydroxybenzoic, hydroxycinnamic acids; Flavonoids; Phenolic terpenes	i) MeOH ii) 1:100 (dried) (*w*/*v*)/1:12.5 (fresh) (*w*/*v*)	i) 10 min ii) n/a	i) Centrifugation (2000 rpm, 10 min); Residue re-extraction (initial conditions); Combination of supernatants; Evaporation (40 °C, Final Volume: 5 mL); Dilution to 10 mL with MeOH ii) n/a	[14]
i) *Origanum vulgare* ii) Herb sample from 2 different sources, dried	Solid-liquid extraction aided by shaking (Soluble, Bound extracts)	Hydroxycinnamic, hydroxybenzoic acids;Phenolic monoterpenes (Soluble extracts) Hydroxycinnamic, hydroxybenzoic acids (Bound extracts)	i) 80% aqueous MeOH (Soluble extracts); 2 M sodium hydroxide (NaOH) (Bound extracts) ii) 1:20 (*w*/*v*) (Soluble extracts); n/a (Bound extracts)	i) 24 h (Soluble extracts); 4 h (Bound extracts) ii) ambient	i) Soluble extracts: Centrifugation (2000 g, 30 min, Ambient T); Supernatant and soluble fraction collection Bound extracts: pH 2.0 with 6 M HCl; Centrifugation (2000 g, 30 min, ambient T); Collection of supernatant; Extraction (15 mL 1:1 (*v*/*v*) Diethylether: AcOEt-three times); Evaporation of organic layers (30 °C); Dissolution to 10 mL with 80% aqueous MeOH ii) n/a	[13]
i) Sicilian *Origanum vulgare* ssp. *hirtum*, *Rosmarinus officinalis,* *Thymus capitatus* L. ii) Dried-aerial parts, flowering season samples from various sites	Solid-liquid extraction (Nonvolatile fraction); Hydrodistillation (Volatile fraction)	Flavonoids (flavones, flavanones) (Nonvolatile fraction); Phenolic terpenes (Volatile fraction)	i) AcOEt and EtOH (Nonvolatile fraction); n/a (Volatile fraction) ii) 1:6.7 (*w*/*v*) (3 times) (Nonvolatile fraction); n/a (Volatile fraction)	i) Overnight in the dark (Nonvolatile fraction); 3 h (Volatile fraction) ii) ambient	i) Nonvolatile fraction: Storage: 4 °C, N_2_-rich atmosphere; Analysis concentration: Dissolution of 10–20 mg of each sample in MeOH (1.5 mL); Filtration. Volatile fraction: (According to European Pharmacopoeia); Drying with sodium sulfate anhydrous (Na_2_SO_4_); Storage: under N_2_ ii) Nonvolatile fraction: Defatting with *n*-hexane; 30 g dried, grounded aerial parts/200 mL; 3 times	[61,62,63]
i) *Thymus serpyllum* ii) Whole-dried	Solid-liquid extraction (Phenolic fraction); Purge & Trap (N_2_, 500 mL N_2_/min) followed by SPE (Volatile fraction)	Flavonoids; Phenolic acids; Phenolic terpenes (monoterpenes)	i) 75% aqueous MeOH (Phenolic fraction); adsorbent: Lichrolut EN (Volatile fraction) ii) 1:4 (*w*/*v*) (Phenolic fraction); 3 g/200 mg (Volatile fraction)	i) 2hr (Phenolic fraction); 90 min (Volatile fraction) ii) n/a	i) Phenolic fraction: Residue washing (5 mL of 75% aqueous MeOH); Combination of extracts; Filtration; Vacuum evaporation (20 °C). Volatile fraction: Elution (Dichloromethane); Dehydration (Anhydrous Sodium Sulphate); Concentration (5 mL, Snyder column, 40 °C); Re-concentration to 0.5 mL (N_2_); Filtration ii) n/a	[64]
i) *Mentha australis* R. Br ii) Fresh leaves and stems	Solid-liquid extraction following sonication	Phenolic acids; Flavonoids (flavanone glycosides)	i) 80% aqueous MeOH ii) 1:20 (*w*/*v*)	i) 10 min, 2 h; overnight ii) 4 °C	i) Extraction 1: Centrifugation (10,000 g, 15 min). Extraction 1, 2, 3: Combination of supernatants; Solvent evaporation (vacuum rotary evaporator, 40 °C) ii) Purification: Glass column (25 × 300 mm i.d.); 50 mL extract; Addition of Amberlite resin; Washing with H_2_O; Elution with 80% aqueous MeOH; Vacuum evaporation (40 °C); Lyophilization (−109 °C, 0.015 k Pa); Analysis concentration: 1 mg (lyophilized, purified) extract/mL MeOH	[65]
i) 3 species of *Salvia* ii) Aerial parts, dried, pulverized	Solid-liquid extraction of the residue obtained after removal of lipophilic substances	Flavonoids (flavones, flavone glycosides)	i) Hot H_2_O (~90 °C) ii) 1:40 (*w*/*v*)	i) Left to reach ambient (T) ii) n/a	i) Partitioning (3 × 100 mL AcOEt, 3 × 100 mL *n*-butanol); Combination of organic phases; Drying (anhydrous magnesium sulfate); Drying (rotary evaporator, 40 °C; Dissolution to 3 mL with MeOH ii) Lipophilic content removal: Shaking (5 g of pulverized sample in *n*-hexane (100 mL), 30 °C, 2 h); Filtration; Stirring overnight (30 °C, 100 mL MeOH: dichloromethane 1:1); Filtration; Drying (rotary evaporator, 40 °C)	[66]
i) *Rosmarinus officinalis* ii) Leaves from 20 geographical zones	Microwave assisted extraction (MAE); two pre-heating steps (160 and 320 W); two extraction cycles (800 W)	Flavonoids; Phenolic diterpenes	i) 70% aqueous MeOH ii) 1:12.5 (*w*/*v*)	i) Each pre-heating step:1 min; Heating gaps: 15 s; Each extraction cycle: 5 min ii) n/a	i) Combination of extracts (two extraction cycles); Filtration; Evaporation (rotary evaporator); Analysis concentration: 800 μg/mL in 50% aqueous MeOH; Filtration ii) n/a	[67]
i) (a) *Origanum majorana*; (b) *Mentha pulegium*; (c) *Lavandula officinalis* ii) (a) Leaves and aerial parts; (b) Flowers; (c) Leaves, dried, milled	MAE (500 W)	Flavonoids Hydroxycinnamic, hydroxybenzoic acids	i) 60 and 80% aqueous MeOH, EtOH and acetone ii) 1:15 (*w*/*v*)	i) 15 min ii) 80 °C	i) Irradiation process: 3 min heating for reaching 80 °C, 3 min for balancing at 80 °C, 5 min for cooling; Filtration ii) n/a	[68]
i) *Rosmarinus officinalis*; *Salvia officinalis*; *Origanum vulgare*; *Thymus vulgaris* ii) Leaves, or herbalmix, or as ingredients in chimichurri sauce	Supercritical fluid extraction—carbondioxide (SFE-CO_2_); Soxhlet Low Pressure Solvent Extraction (LPSE) (17.3 g/min); Ultrasound assisted extraction (UAE) (40 kHz;1 bar; 20 g of CO_2_/g raw material solvent)	Phenolic terpenes (diterpenes)	i) CO_2_ for SFE; EtOH for Soxhlet LPSE and UAE; ii) n/a for SFE and UAE; 1:30 for Soxhlet and UAE.	i) 6 h ii) 40 °C for SFE and S; n/a for Soxhlet; 50 °C for UAE	i) n/a for SFE; Vacuum evaporation (40 °C) for Soxhlet and UAE ii) n/a	[69]
i) 10 *Salvia* species ii) Plant material, dried	SFE-CO_2_ (45 MPa, CO_2_: 2 L/min) Accelerated solvent extraction (ASE) (10.3 MPa)	Flavonoids; Phenolic terpenes; Hydroxybenzoic, hydroxycinnamic acids; Phenolic acids (caffeic acid derivatives)	i) CO_2_ (99.9%) for SFE; 96% EtOH, followed by H_2_O for ASE ii) n/a for SFE; 3:1 in diatomaceous earth for ASE	i) 60 min. (SFE-CO_2_); 30 min. (ASE) ii) 60°C for SFE; 140 °C for ASE	i) ASE: EtOH evaporation; Lyophilization of H_2_O extracts ii) n/a	[3]
i) *Salvia officinalis, Thymus serpyllum*, *Origanum vulgare, Melissa officinalis* ii) Plant raw material, grounded	Heating; MAE; Sonication; Subcritical extraction	Phenol carboxylic; Cinnamic acids; Flavonoids; Phenolic terpenes (diterpenes)	i) 70% aqueous EtOH ii) 1:50 (*w*/*v*)	i) n/a ii) n/a	i) (According to the Russian State Pharmacopoeia, FS.2.5.0051.15). Centrifugation; Filtration ii) n/a	[70]

^1^ Purification/Clean-up step took place either in parallel or subsequently to the extraction of (poly) phenolic/bioactive compounds.

**Table 2:** Recent applications of conventional and hyphenated chromatographic methods for phenolic constituents in *Lamiaceae* species.

i) *Lamiaceae* Species ii) Plant Part	Polyphenols Analysed ^1^	Chromatography	Detection System	Chromatographic Conditions and Method Validation Results	Reference(s)
i) *Thymus vulgaris* ii) Aerial parts	C17, **C21**	Capillary electrophoresis (CE)	UV-diode array detector (DAD)	Capillary: Fused silica (66 cm length, 58 cm effective length, 75 mm internal diameter (i.d.)) Capillary (T): 23 °C Background electrolyte solution: borax buffer (20 mM, pH 10.0): 90% MeOH Driving voltage: 23 kV limit of detection (LOD) for C17: 0.53 μg/mL, LOD for C21: 1.05 μg/mL limit of quantification (LOQ) for C17: 1.41 μg/mL, LOQ for C21: 2.98 μg/mL correlation/determination coefficient (*R*^2^) for C17: 0.9990, (*R*^2^) for C21: 0.9999	[47]
i) *Melissa officinalis* ii) Fresh herbs or leaves from 12 manufacturers	C57, C59, **C63**, C64, C66, C67	High performance liquid chromatography (HPLC)	UV/Vis	Column: Hypersil GOLD C18 (250 mm × 4.6 mm i.d., 5.0 µm particle size (p.s.)) (T): 30 °C Eluents: (A) 0.05% trifluoroacetic acid (TFA) in MeOH; (B) 0.05% TFA in H_2_O Run (t): 35 min LOD: 0.16–0.51 µg/mL, LOQ: 0.42–1.54 µg/mL, (*R*^2^): ≥0.9089	[49]
i) *Mentha pulegium*, *Nepeta nuda* ii) Aerial parts	**C17**, C19, C21, C22, C33, C41, C44, C45, C46, **C57**, C59, C64	HPLC	UV-photodiode array (PDA) detector	Column: LiChrospher 100 RP C18 endcapped (250 mm × 4.6 mm i.d., 5.0 µm p.s.) Eluents: (A) H_2_O containing 0.02% phosphoric acid and (B) MeCN Run (t): 70 min	[46]
i) *Origanum vulgare* ii) Herb sample from different sources, dried	C15, C16, C34, C36, C38, C55, C56, **C57**, C58, C59, C61, C63, **C66**, C69, C71, **C75**	HPLC	DAD	Column: Zorbax SB-Aq (250 mm × 4.6 mm i.d., 5.0 µm p.s.) Eluents: (A) 0.5% formic acid in H_2_O; (B) MeOH Run (t): 95 min	[13]
i) *Salvia officinalis*, *Thymus serpyllum*, *Origanum vulgare*, *Melissa officinalis* ii) Plant (raw) material	C22, **C23**, C46, C57, C58, C59, C61, **C63**, C64, C65, C66, C68, C69, C70, C78	HPLC	DAD	Column: Phenomenex Luna C18 (250 mm × 4.6 mm i.d., 5.0 µm p.s.) (T): 40 °C Eluents: (A) MeCN; (B) 1% acetic acid in H_2_O Run (t): 35 min LOD: 0.10–0.30 µg/mL, (*R*^2^): ≥ 0.999	[70]
i) *Origanum vulgare* ssp. *hirtum*, *Thymus capitatus*, *Satureja thymbra*, *Melissa officinalis*, *Rosmarinus officinalis* ii)Aerial parts, dried, grounded leaves and flowers	C1, C17, C21, C34, C36, **C37**, C40, C46, C48, C50, C57, C58, C59, C60, C61, **C63**, C64, C66, C67, C68, C69, C70, C74, **C75**	RP-HPLC	DAD	Column: Nucleosil 100 C18 (250 mm × 4.6 mm i.d., 5.0 µm p.s.) (T): 30 °C Eluents: (A) 1% acetic acid in H_2_O; (B) MeCN; (C) MeOH Run (t): 55 min LOD: 0.002–0.16 µg/mL, LOQ: 0.01–0.48 µg/mL, (*R*^2^): ≥ 0.9961	[50]
i) (a) *Rosmarinus officinalis*, *Origanum majorana*, *Origanum vulgare*; (b) *Ocimum basilicum, Mentha spicata*, *Thymus vulgaris*; (c) *Mentha* x *piperita*, *Thymus* x *citriodorus* ii) (a) Fresh; (b) Dried; (c) Organic-dried	C5, **C16**, C36, C40, C50, C55, C58, C59, C61, C62, **C63**, C64, C66, C69, **C74**, C75, C78, **C79**	ultra-high-performance liquid chromatography (UHPLC)	DAD	Column: Acquity ‘ethylen e bridged hybrid (BEH C18 (50 mm × 2.1 mm i.d., 1.7 μm p.s.) with an Acquity UHPLC BEH C18 VanGuard pre-column (5 mm × 2.1 mm i.d., 1.7 μm p.s.) (T): 20 °C Eluents: (A) 0.1% acetic acid in H_2_O; (B) 0.1% Acetic acid in MeCN Run (t): 30 min LOD: 0.01–0.38 µg/mL, LOQ: 0.04–1.14 µg/mL, (*R*^2^): ≥0.9990	[14]
i) *3Mentha* ssp. ii) Dried and powdered leaves	C1, C4, C7, C21, **C28**, C46, C48, C57, C58, C59, **C63**, **C64**, C66, C70	HPLC	DAD	Column: Grace^TM^ Alltech^TM^ Alltima^TM^ C18 (250 mm × 4.6 mm i.d., 5.0 µm p.s.) (T): 40 °C Eluents: (A) MeCN: H_2_O: formic acid (19:80:1); (B) MeCN: MeOH: formic acid (59:40:1) Run (t): 45 min	[57]
i) (a) 11 species of *Mentha*, (b) 2 Mixtures of *Mentha* species ii) (a) Plant material; (b) Finished Pharmaceutical products (2 Manufactures)	C1, C3, C10, C17, C21, C22, C28, C32, C46, C57, C63	two-dimensional micro-thin layer chromatography (2D-mTLC)	UV	Plate: HPTLC CNF 254 (10 cm × 10 cm, in 5 cm × 5 cm squares) Derivatization reagent: Naturstoff reagent 1st Condition: Non-aqueous eluent: 40% propan-2-ol in n-heptane; Aqueous eluent: 30% MeCN 2nd Condition: Non-aqueous eluent: 80% AcOEt in n-heptane; Aqueous eluent: 50% aqueous MeOH Sample quantity: 5 µL Conditioning: 20–30 min	[48]
i) *Thymus vulgaris*; *Salvia officinalis* ii) Aerial parts	C17, C19, C21, C22, C40, C45, C46, C57, C63, C64, C74	TLC	UV	Plate: Pre-coated silica gel TLC plates Si60 F254 Derivatization reagent: natural products-polyethylene glycol reagent (NP/PEG); 2,2-diphenyl-1-picrylhydrazyl radical (DPPH•_ in 0.2% in MeOH; Wavelength: 366 nm Eluents: For flavonoid aglycones: toluene: diethyl ether: acetic acid (60:40:10); For flavonoid glycosides: AcOEt: acetic acid: formic acid: H_2_O (100:11:11:26); For phenolic acids: chloroform: ethyl acetate: acetone: formic acid (40:30:20:10)	[58]
		HPLC	DAD; MS in positive ion mode	Column: Zorbax Eclipse Plus PAH C18 (100 mm × 2.1 mm i.d. × 1.8 µm p.s.) Eluents: (A) 0.1% formic acid in H_2_O; (B) 0.1% formic acid in MeCN Run (t): 30 min	
i) *Origanum vulgare*, *Ocimum basilicum, Rosmarinus officinalis*, *Origanum majorana*, *Thymus vulgaris*, *Satureja hortensi*s ii) Commercial, dried, grounded leaves	**C63**, **C81**	HPLC	UV-DPPH•; electrospray ionization (ESI)-MS in negative and positive ion mode	Column: Synergi Max-RP C12 (250 mm × 4.6 mm i.d., 4.0 µm p.s.) (T): 25 °C Eluents: (A) 0.05% TFA in H_2_O; (B) 60% MeCN in MeOH Run (t): 60 min	[2]
i) *Rosmarinus officinalis*; *Origanum vulgare*; *Salvia officinalis*; *Thymus vulgaris*; *Origanum vulgare* ii) Leaves, or herbal mix, or as ingredients in chimichurri sauce	**C63**, **C76** *, **C78**, **C79** *, **C80** *	HPLC	PDA	Column: Kinetex Polar C18 (250 mm × 4.6 mm i.d., 2.6 µm p.s.) (T): 55 °C Eluents: (A) 0.1% acetic acid in H_2_O; (B) 0.1% acetic acid in MeCN Run (t): 10 min LOD: 0.25 μg/mL, LOQ: 1.0 μg/mL, (*R*^2^): ≥0.9998	[69]
		UHPLC	MS in negative ion mode	Column: Acquity UHPLC BEH C18 (50 mm × 2.1 mm i.d., 1.7 µm p.s.) (T): 55 °C Eluents: (A) 0.1% acetic acid in H_2_O; (B) 0.1% acetic acid in MeCN Run (t): 10 min	
i) 3 species of *Savlia* ii) Aerial parts, dried	Tentative identification only	LC	DAD-ESI-MS in positive ion mode	Column: Phenomenex Superspher 100 RP C18 (125 mm × 4.6 mm i.d. × 4.0 µm p.s.) (T): 40°C Eluents: (A) 2.5% acetic acid in H_2_O; (B) MeOH Run (t): 30 min	[66]
i) *Satureja montana* ssp. *kitaibelii* ii) Aerial parts of wild plant, air-dried	C17, C40, C46, C57, C59, **C64**, C69, C73	HPLC	DAD–ESI-time-of flight (TOF)–MS	Column: Agilent Poroshell 120 C18 endcapped (100 mm × 4.6 mm i.d., 2.7 µm p.s.) (T): 25 °C Eluents: (A) 1% acetic acid in H_2_O; (B) MeCN Run (t): 36 min LOD: 0.187–2.471 μg/mL, LOQ: 0.623–8.238 μg/mL, (*R*^2^): ≥0.9983	[55]
i) Sicilian *Origanum vulgare* ssp. *hirtum, Rosmarinus officinalis, Thymus capitatus* L. ii) Dried-aerial parts, flowering season, samples from various sites	C1, C9, C13, **C14** *, C17, C21, C57, **C63**, **C74** *, **C75** *, **C78** *, **C79** *, **C80** *	HPLC	PDA/ESI-MS in positive and negative ion mode	Column: Phenomenex Luna C18 endcapped (250 mm × 4.6 mm i.d., 5.0 µm p.s.) (T): 25 °C Eluents: (A) 1% formic acid in H_2_O; (B) MeCN Run (t): 64 min	[61,62,63]
		GC	flame ionization detector (FID)/MS	Column: SPB-5 capillary (15 m length × 0.1 mm i.d. × 0.15 μm thickness) Injection: Split ratio (1:200)Oven (T): 60 °C for 1 min, linearly rising from 60 to 280 °C with a rate of 10 °C/min, 280 °C for 1 min	
i) (a) *Origanum majorana*; (b) *Mentha pulegium*; (c) *Lavandula officinalis* ii) (a) Leaves and aerial parts; (b) Flowers; (c) Leaves, dried, milled	C1, C17, C34, C40, C46, C48, C51, C52, C57, C58, C59, C60, **C63**, C66, C67, C68, C69	UHPLC	DAD; ESI-tandem mass spectrometry (MS/MS) in negative ion and multiple reaction monitoring (MRM) mode	Column: Acquity UHPLC BEH C18 (100 mm × 2.1 mm i.d., 1.7 µm p.s.) (T): 30 °C Eluents: (A) 1% formic acid in H_2_O; (B) 1% formic acid in MeOH Run (t): 12 min LOD: 0.02–5.52 ng/mL, LOQ: 0.06–18.20 ng/mL, linear regression (r): ≥0.9988	[68]
i) *Thymus* x *citriodorus* ii) Mixture of leaves and stems, dried	C2, C8, C19, C20, **C20** *, C22, **C23** *, C24, **C63**	RP-HPLC	DAD; ESI–MS and multi-stage mass spectrometry (MS^n^) in negative ion mode; nuclear magnetic resonance (NMR)	Column: Nucleosil C18 endcapped (250 mm × 4.0 mm i.d., 5.0 µm p.s.) (T): 30 °C Eluents: (A) 0.1% formic acid in H_2_O; (B) MeCN Run (t): 30 min LOD: 1.0–12.4 µg/mL, LOQ: 3.0–37.7 µg/mL, (*R*^2^): ≥0.9984	[59]
i) *Origanum majorana* ii)Commercially produced, dried/grounded	C17, C22, C37, C40, **C62** *, **C63**, C66	LC	ESI-MS/MS in negative ion mode; ^1^H NMR	Column: Atlantis T3 C18 (100 mm × 2.1 mm i.d. × 3 µm p.s.) (T): 40 °C Eluents: (A) 0.5% formic acid in H_2_O; (B) 0.5% formic acid in (MeCN: MeOH, 50:50) Run (t): 26 min	[60]
i) *Rosmarinus officinalis*; *Origanum vulgare*; *Origanum majorana*; *Thymus vulgaris* ii) Dried, grounded	C34, C36, C40, C57, C58, C59, **C63**, C67, C64, **C69**, C70	LC	PDA; ESI-linear ion trap quadrupole (LTQ)-Orbitrap-MS in negative ion mode	Column: Atlantis T3 RP C18 (100 mm × 2.1 mm i.d., 3 µm p.s.) (T): 25 °C Eluents: (A) 0.1% formic acid in H_2_O; (B) 0.1% formic acid in MeCN Run (t): 36 min LOD: 1.7 × 10^−3^–8.9 × 10^−3^ µg/g DW	[11,51]
i) *Rosmarinus officinalis* ii) Leaves from 20 differentgeographical zones	C6, C22, C25, C26, **C27** *, **C35** *, C37, **C63**, **C67**, **C78**, C79	HPLC	ESI-QTOF-MS and MS/MS in negative ion mode	Column: Zorbax Eclipse Plus C18 (150 mm × 4.6 mm i.d., 1.8 µm p.s.) (T): ≈20–25 °C Eluents: (A) 0.1% formic acid in H_2_O; (B) MeCN Run (t): 30 min LOD: 0.014–0.24 µg/mL, LOQ: 0.04–0.8 µg/mL, (*R*^2^): ≥0.9803	[67]
i) *Mentha pulegium*, *Origanum majorana* ii) Aerial parts	C13, C17, **C21**, **C37**, **C54**	RP-UHPLC	ESI-QTOF-MS and MS/MS in negative ion mode	Column: Zorbax Eclipse Plus C18 (150 mm × 4.6 mm i.d., 1.8 µm p.s.) (T): 25 °C Eluents: (A) 0.5% acetic acid in H_2_O; (B) MeCN Run (t): 33 min	[52]
i) *Mentha spicata* ii) Commercial extract	C3, C31, C46, **C63**, C64, C65, C82, C83, C84	UHPLC	ESI-MS^n^ in negative ion mode	Column: BlueOrchid C18 (50 mm × 2.0 mm i.d., 1.8 µm p.s.) (T): 30 °C Eluents: (A) 0.1% formic acid in H_2_O; (B) 0.1% formic acid in MeCN Run (t): 20 min	[56]
i) *Thymus serpyllum* ii) Whole-dried	C7, **C21**, **C22** *, C39, C46, C57, C63, C64, C66, C69, **C74**, C75	RP-LC	DAD-ESI-MS/MS FID; mass selective detector (MSD);	Column: Phenomenex RP C18 (250 mm × 4.6 mm i.d. × 5.0 µm p.s.) (T): 25 °C Flow rate: 0.7 mL/min Eluents: (A) 1% formic acid in H_2_O; (B) (MeCN/Solvent A) (60:40) Run (t): 106 min	[64]
		GC	mass spectrometry-olfactometry (MS-O)	Column: DB-Wax column (30 m length × 0.25 mm i.d. × 0.5 μm thickness) Injection: Pulsed splitless (40 psi; 0.5 min) Injector (T): 270 °C FID (T): 280 °C Oven (T): 250 °C for 10 min (50–250 °C with a rate of 4 °C/min)	
i) *Rosmarinus officinalis* ii) Branded extract rich in carnosic acid	C4, C18, C46, C57, C63, C76, C77, **C78**, C79	UHPLC	ESI-MS^n^ in negative ion mode	Column: XSelect HSS T3 C18 (50 mm × 2.1 mm i.d., 2.5 µm p.s.) (T): 30 °C Eluents: (A) 0.1% formic acid in H_2_O; (B) 0.1% formic acid in MeCN Run (t): 35 min	[53]
i) *Mentha australis* R. Br ii) Fresh leaves and stems	C1, C4, C5, **C11** *, C17, C57, **C63**, C64	HPLC	PDA	Column: Phenomenex Luna C18 endcapped (250 mm × 4.6 mm i.d., 5.0 µm p.s.) (T): 30 °C Eluents: (A) 2.5% acetic acid in H_2_O; (B) MeCN Run (t): 34 min Fraction collection (major peaks): Column: Phenomenex Luna 10 μm C18 (250 mm × 15 mm) Eluents: Similar to HPLC Run (t): Similar to HPLC	[65]
		LC	Heated electrospay ionization (HESI)/atmospheric pressure chemical ionization	Similar conditions with HPLC. LOD: 0.25 ng	
		LC	(APCI)-MS/MS positive and negative ion mode; NMR HESI/APCI-high resolution mass spectrometry (HRMS) in positive and negative ion mode	Column: Phenomenex Synergi Hydro-RP C18 (250 mm × 1.0 mm i.d., 4.0 µm p.s.) (T): 45 °C Eluents: (A) 5 mM ammonium formate in H_2_O (pH 7.4) (B) 5 mM ammonium formate in 90% aqueous MeOH (pH 7.4) Run (t): 19 min LOD: 0.625 ng	
i) 6 *Ocimum* ssp. ii) Leaves, dried, grounded	C17, C21, C40, C43, **C46**, C48, C49, C55, C57, C59, C60, **C63**, C64, C66, C69	UHPLC	ESI-hybrid linear ion trap (QqQLIT) in negative ion mode	Column: Acquity UHPLC BEH C18 (50 mm × 2.1 mm i.d., 1.7 µm p.s.) (T): 50 °C Eluents: (A) 0.1% formic acid in H_2_O; (B) 0.1% formic acid in MeCN Run (t): 13 min LOD: 0.041–0.357 ng/mL, LOQ: 0.124–1.082 ng/mL	[54]
i) 10 *Salvia* species ii) Plant material, dried	C17, **C20**, C21, C23, C29, C30, C42, C45, C47, C53, **C57**, **C63**, C71, C78, C79	HPLC	UV-DPPH•-MS PDA	Column: Discovery HS C18 (250 mm × 4.6 mm i.d., 5.0 µm p.s.) Flow rate: 0.8 mL/min Injection Volume: 20 µL Eluents: (A) 0.1% formic acid in H_2_O; (B) MeOH Run (t): 60 min	[3]
		UHPLC	ESI-QTOF, triple quadrupole-spectrometer (TQ-S) in negative mode	Column: Acquity UHPLC BEH C18 (100 mm × 2.1 mm i.d., 1.7 µm p.s.) (T): 40 °C Eluents: (A) 0.1% formic acid in H_2_O; (B) MeCN Run (t): 11 min LOD: 1.67–13.39 µg/mL, LOQ: 5.56–44.65 µg/mL	

^1^ The reference analytical standards employed in each research. Note: The letter C followed by numbers correspond to the chemical structures and names that are given in Figure S1 (a, b, c, d, and e). The ‘bold’ compounds represent the most abundant polyphenols in the species analysed. The ‘bold’ compounds followed by *, represent the most abundant polyphenols that were tentatively quantified in the species analysed.

## Supplementary Materials

The following are available online at http://www.mdpi.com/2223-7747/7/2/25/s1, Figure S1 (a, b, c, d, e): The chemical structures of the analytical standards or the most abundant polyphenols in the analysed species, Table S1: (Poly) phenolic compounds identified for the first time in the literature cited in Table 2.

## Abbreviations

The following abbreviations are used in this Manuscript:

AcOEt	ethyl acetate
APCI	atmospheric pressure chemical ionization
ASE	accelerated solvent extraction
BHA	butylated hydroxyanisole
BHT	butylated hydroxytoluene
CE	capillary electrophoresis
CO_2_	carbon dioxide
DAD	diode array
DPPH•	2,2-diphenyl-1-picrylhydrazyl radical
ESI	electrospray ionization
EtOH	ethanol
FC	flash chromatography
FID	flame ionization detection
GC	gas chromatography
hr	hours
H_2_O	water
HCl	hydrochloric acid
HESI	heated electrospay ionization
HPLC	high-performance liquid chromatography
HRMS	high resolution mass spectrometry
i.d.	internal diameter
LC	liquid chromatography
LOD	limit of detection
LOQ	limit of quantification
LPSE	low pressure solvent extraction
LTQ	linear ion trap quadrupole
QqQLIT	hybrid linear ion trap
MAE	microwave assisted extraction
ME	matrix effects
MeCN	acetonitrile
MeOH	methanol
MRM	multiple reaction monitoring
MS	mass spectrometry
MS^n^	multi-stage mass spectrometry
MS/MS	tandem mass spectrometry
MSD	mass selective detector
MS-O	mass spectrometry-olfactometry
*m/z*n/a	mass-to-charge ratio not available
NaOH	sodium hydroxide
N_2_	Nitrogen
NMR	nuclear magnetic resonance
NP/PEG	natural products-polyethylene glycol reagent
O_2_	Oxygen
P & T	purge and trap
PDA	photodiode array
p.s.	particle size
r	linear regression
R^2^	correlation/determination coefficient
RP	reversed phase
RSC	radical scavenging capacity
SFE	supercritical fluid extraction
SIM	selected ion monitoring mode
SPE	solid phase extraction
mTLC	micro-thin layer chromatography
T	temperature
t	Time
TFA	trifluoroacetic acid
TLC	thin layer chromatography
TOF	time-of-flight
TQS	triple quadrupole spectrometer
UAE	ultrasound-assisted extraction
UHPLC	ultra-high performance liquid chromatography
UV	ultraviolet
Vis *v*/*v* *w*/*v*	visible volume/volume weight/volume
2D	two dimensional
