# Supplementary material for: Qualitative and Quantitative Analysis of Polyphenols in Lamiaceae Plants—A Review
Source: Plants (Basel). 2018 Mar 26;7(2):25. doi: 10.3390/plants7020025 (PMC6027318; doi:10.3390/plants7020025)
Supplement: Supplementary file 1 [file plants-07-00025-s001.pdf]

plants

Qualitative and quantitative analysis of polyphenols in *Lamiaceae* herbs – A review

Katerina Tzima[1, 2], Nigel P. Brunton[2] and Dilip K. Rai[1, *]

[1] Department of Food BioSciences, Teagasc Food Research Centre Ashtown, Dublin, D15 KN3K, Ireland
[2] UCD Institute of Food and Health, University College Dublin, Belfield, Dublin 4, Ireland

Supplementary Materials

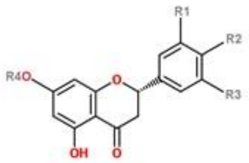

	Flavanones	R1	R2	R3	R4
**C1**	naringenin	H	OH	H	H
**C2**	naringenin-7-O-glucoside	H	OH	H	glc **a**
**C3**	narirutin	H	OH	H	rut **b**
**C4**	hesperidin	OH	OCH_3	H	rut **b**
**C5**	hesperetin	OH	OCH_3	H	H
**C6**	neohesperidin	H	OCH_3	OH	neo **c**
**C7**	naringin	H	OH	H	neo **c**
**C8**	eriodictyol-7-O-glucoside	OH	OH	H	glc **a**
**C9**	eriodictyol	OH	OH	H	H
**C10**	eriocitrin	OH	OH	H	rut **b**
**C11**	neoponcirin	H	OCH_3	H	rut **b**

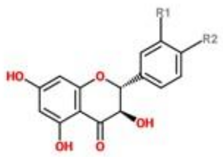

	Flavanolons	R1	R2
**C12**	dihydrokaempferide	H	CH_3
**C13**	taxifolin	OH	OH
**C14**	taxifolin-di-O-glucoside	glc **a**	glc **a**

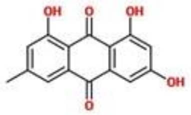

Resins

C15 emodin

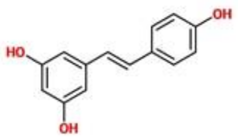

Stilbenoids

C16 resveratrol

a. glucoside

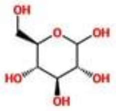

b. rutinoside

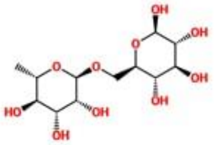

c. neohesperidoside

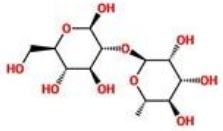

(**a**)

Figure S1. (**a**) The chemical structures of the analytical standards or the most abundant polyphenols in the analysed species.

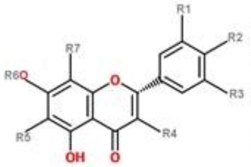

	Flavones	R1	R2	R3	R4	R5	R6	R7
**C17**	apigenin	H	OH	H	H	H	H	H
**C18**	apigenin-8-C-glucoside	H	OH	H	H	H	H	O-glc **a**
**C19**	apigenin-7-O-glucoside	H	OH	H	H	H	glc **a**	H
**C20**	apigenin-7-O-glucuronide	H	OH	H	H	H	gluc **d**	H
**C21**	luteolin	OH	OH	H	H	H	H	H
**C22**	luteolin-7-O-glucoside	OH	OH	H	H	H	glc **a**	H
**C23**	luteolin-7-O-glucuronide	OH	OH	H	H	H	gluc **d**	H
**C24**	chrysoeriol-7-β-O-glucoside	H	OH	OCH_3	H	H	glc **a**	H
**C25**	genkwanin	H	OH	H	H	H	CH_3	H
**C26**	homoplantaginin	H	OH	H	H	OCH_3	glc **a**	H
**C27**	nepetrin	H	OH	OH	H	OCH_3	glc **a**	H
**C28**	diosmin	H	OCH_3	OH	H	H	rha **e**	H
**C29**	diosmetin	OH	OCH_3	H	H	H	H	H
**C30**	3',4',5,7-tetrahydroxy-3-methoxyflavone	H	OH	OH	OCH_3	H	H	H
**C31**	orientin	OH	OH	H	H	H	H	O-glc **a**
**C32**	isorhoifolin	H	OH	H	H	H	rut **b**	H
**C33**	vitexin-2''-O-rhamnoside	H	OH	H	H	H	H	O-rha **e**

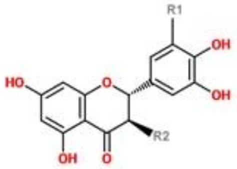

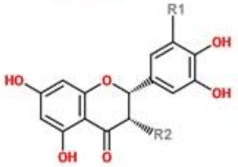

	Flavan-3-ols	R1	R2
**C34**	catechin	H	OH
**C35**	gallocatechin	OH	OH
**C36**	epicatechin	H	OH
**C37**	(-)-epigallo catechin	OH	OH

Phenolic aldehydes

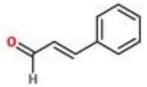

C38 cinnamaldehyde

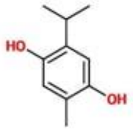

C39 *p*-cymene-2,5-diol

d. glucuronide

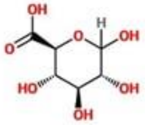

e. rhamnoside

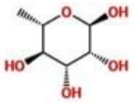

(**b**)

Figure S1. (**b**) The chemical structures of the analytical standards or the most abundant polyphenols in the analysed species.

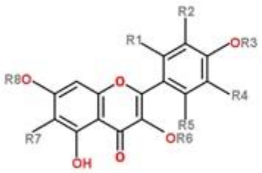

	**Flavonols**	**R1**	**R2**	**R3**	**R4**	**R5**	**R6**	**R7**	**R8**
**C40**	quercetin	H	OH	H	H	H	H	H	H
**C41**	quercetin dihydrate (·$2H_2O$)	H	OH	H	H	H	H	H	H
**C42**	quercetin-3-O-glucuronide	H	OH	H	H	H	gluc **d**	H	H
**C43**	quercetin-3,4'-diglucoside	H	OH	glc **a**	H	H	glc **a**	H	H
**C44**	isoquercitrin	H	OH	H	H	H	glc **a**	H	H
**C45**	hyperoside	H	OH	H	H	H	gal **f**	H	H
**C46**	rutin	H	H	H	OH	H	rut **b**	H	H
**C47**	rutin hydrate (·$3H_2O$)	H	H	H	OH	H	rut **b**	H	H
**C48**	kaempferol	H	H	H	H	H	H	H	H
**C49**	kaempferol-3-O-rutinoside	H	H	H	H	H	rut **b**	H	H
**C50**	myricetin	H	OH	H	OH	H	H	H	H
**C51**	morin	OH	H	H	H	H	H	H	H
**C52**	fisetin	H	H	H	OH	H	H	H	H
**C53**	rhamnetin	H	OH	H	H	H	H	H	CH_3
**C54**	jaceidin	H	OCH_3	H	H	H	CH_3	OCH_3	H

Hydroxyphenylpropenes

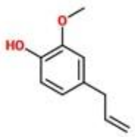

C55 eugenol

Hydroxycoumarins

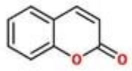

C56 coumarin

f.

galactoside

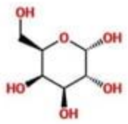

(**c**)

Figure S1. (**c**) The chemical structures of the analytical standards or the most abundant polyphenols in the analysed species.

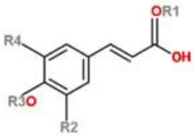

	Hydroxycinnamic acids	R1	R2	R3	R4
**C57**	caffeic acid	H	OH	H	H
**C58**	*p*-coumaric acid	H	H	H	H
**C59**	ferulic acid	H	H	H	OCH_3
**C60**	sinapic acid	H	OCH_3	H	OCH_3

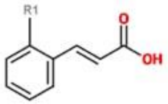

		R1
**C61**	cinnamic acid	H
**C62**	2-hydroxycinnamic acid	OH

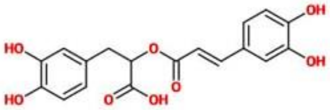

C63 rosmarinic acid

quinic acid

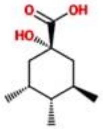

		R1	R2	R3
**C64**	chlorogenic acid	C57	H	H
**C65**	neochlorogenic acid	H	H	C57

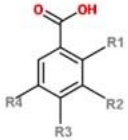

	Hydroxybenzoic acids	R1	R2	R3	R4
**C66**	gallic acid	OH	OH	OH	H
**C67**	syringic acid	OCH_3	OH	OCH_3	H
**C68**	vanillic acid	H	OH	OCH_3	H
**C69**	protocatechuic acid	H	OH	OH	H
**C70**	4-hydroxbenzoic acid	H	OH	H	H

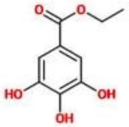

C71 ethyl gallate

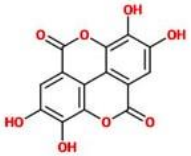

C72 ellagic acid

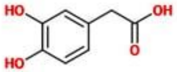

Hydroxyphenylacetic acids

C73 3,4-dihydroxyphenylacetic acid

(**d**)

Figure S1. (**d**)The chemical structures of the analytical standards or the most abundant polyphenols in the analysed species.

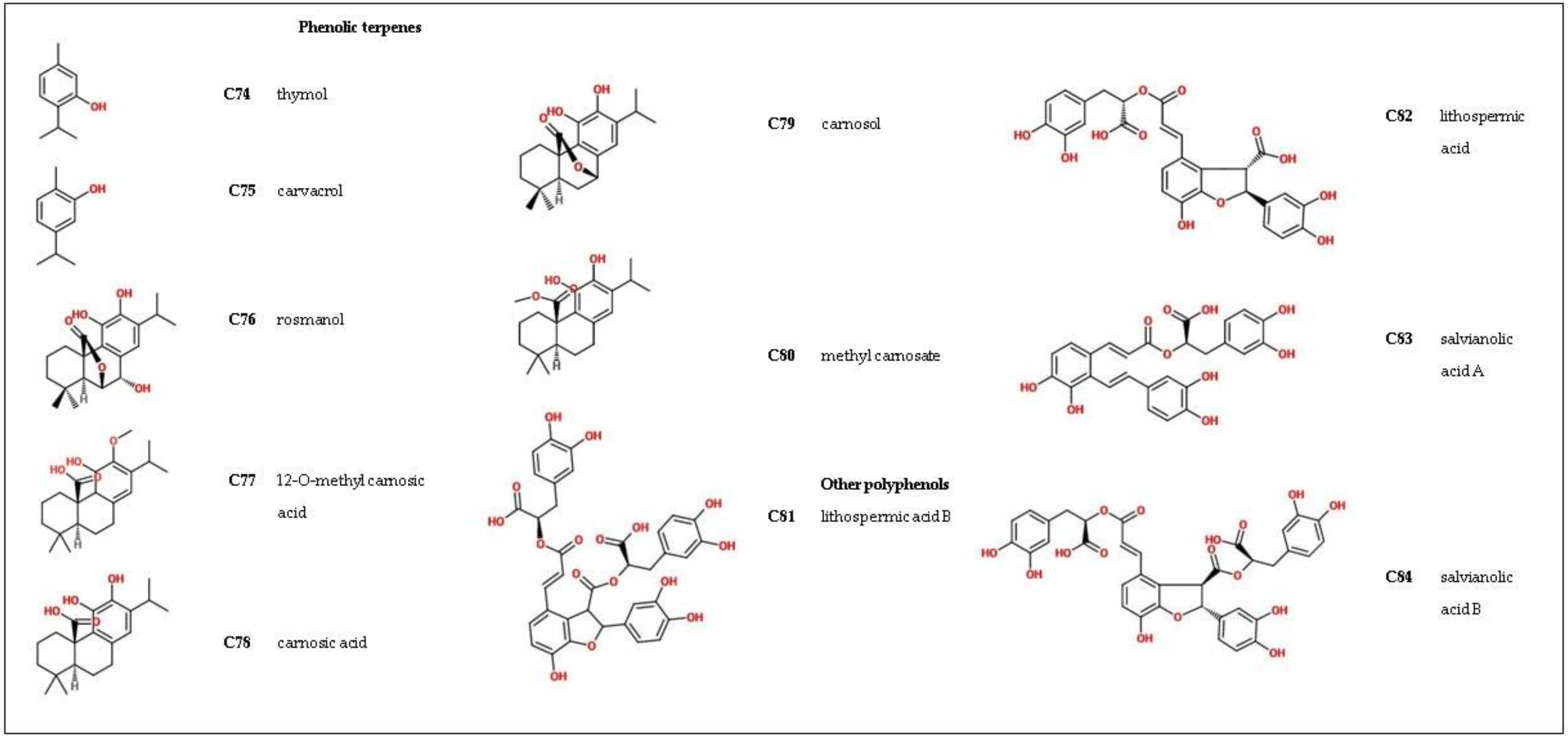

(e)

Figure S1. (**e**) The chemical structures of the analytical standards or the most abundant polyphenols in the analysed species.

Table S1. (Poly) phenolic compounds identified for the first time in the different researches of Table 2.

***Lamiaceae* herbs**	**(Poly) phenolic compounds identified for the first time**	**Referenc e**
Origanum vulgare	1. apigenin-C-hexoside-C-hexoside	[11]
Mentha australis R. Br<br>Identified for the first time in *Mentha* genus.	1. neoponcirin<br>2. biochanin A	[65]
Tunisian *Mentha pulegium*	1. salvianolic acid B<br>2. salvianolic acid E<br>3. isosalvianolic acid B<br>4. salvialonolic acid I<br>5. salvianolic acid H<br>6.lithospermic acid<br>7. luteolin-6,8-C-dihexose<br>8. syringetin<br>9. quercetin,<br>10. dimethyl ether<br>11. 2 jaceidin isomers<br>12. hydroxybenzoic acid hexose isomers<br>13. dihydroxybenzoic acid hexose<br>14.vanillic acid hexose<br>15. syringic acid hexose	[52]
Thymus serpyllum	1. protocatechuic acid<br>2. protocatechuic acid-hexoside<br>3. gallic acid<br>4. rosmarinic acid-glucoside<br>5. apigenin 6, 8-di-C-glucoside<br>6. apigenin O-glucuronide<br>7. luteolin-O-diglucuronide<br>8. naringin<br>9. kaempferol O-glucuronide<br>10. methyl kaempferol O-rutinoside	[64]
Thymus x *citriodorus*	1. luteolin-7-O-glucuronide<br>(first time in *Thymus* species)<br>2. eriodictyol dihexoside with O-glycosidic linkages,<br>(first time in *Thymus* species)<br>3. eriodictyol-O-monohexosides (2)<br>(first time in *Thymus* species)<br>4. quercetagetin dimethyl ether-O-hexoside (1)<br>(first time in *Thymus* species)<br>5. naringenin-O-hexoside (1)<br>(first time in *Thymus* species)<br>6. chrysoeriol-7-β-O-glucoside<br>(first time in *Thymus* species)<br>7. apigenin-7-β-O-glucuronide<br>(first time in *Thymus* x *citriodorus*)	[59]
Thymus vulgaris	1. lithospermic acid B	[2]
Thymus vulgaris	1. sinapic acid-C-hexoside	[11]

Origanum majorana	1. lithospermic acid B	[2]
Tunisian *Origanum majorana*	1.kaempferol-O-glucuronide<br>2. sakuranetin<br>3. salvialonolic acid I<br>4. luteolin-6,8-C-dihexose<br>(first time in *Origanum majorana* species)<br>5. taxifolin<br>(and two isomer derivatives)<br>6. dihydrokaempferide<br>7. luteolin-O-glycoside<br>8. kaempferol-O-sambubioside<br>(not reported in the literature before)<br>9. luteolin glucoside<br>10. syringetin<br>11. quercetin,<br>12. dimethyl ether<br>13. 2 jaceidin isomers<br>14. hydroxybenzoic acid hexose isomers<br>15. dihydroxybenzoic acid hexose<br>16. vanillic acid hexose<br>17. syringic acid hexose	[52]
Satureja hortensis	1. lithospermic acid B	[2]
Rosmarinus officinalis	1. sinapic acid-C-hexoside<br>2. apigenin-C-hexoside-C-hexoside	[11]
Rosmarinus officinalis<br>Branded extract	1. dihydroxy-dimethoxyflavone derivative<br>2. dihydroxy-dimethoxyflavone<br>3. medioresinol derivative<br>4. dihydroxy-dimethoxyflavone<br>5. medioresinol-glucuronide<br>6. isorhamnetin-rutinoside<br>7. hispidulin-rutinoside<br>8. 5,6,7,10-tetrahydro-7-hydroxy rosmariquinone derivative<br>9. isosakuranetin<br>10. carnosic acid hexoside<br>11. 5,6,7,10-tetrahydro-7-hydroxyrosmariquinone derivative<br>12. carnosic acid derivative<br>13. 40-Methoxytectochrysin<br>14. 5,6,7,10-tetrahydro-7-hydroxy rosmariquinone	[53]
Satureja montana ssp. *kitaibelii*	1. quinic acid<br>2. dihydroxybenzoic acid glucoside isomer 1<br>3. dihydroxybenzoic acid glucoside isomer 2<br>4. caffeoylquinic acid isomer 1<br>5. luteolin-7-O-b-glucopyranoside<br>6. chlorogenic acid<br>7. caffeoylquinic acid isomer 3<br>8. caffeoylquinic acid methyl ester<br>9. dihydroferulic acid 4-O-glucuronide/<br>3-methoxy-4,5-dihydroxycinnamic acid glucoside<br>10. quercetagetin 7-β-D-glucoside<br>11. quercetin 3-β-D-glucoside<br>12. acacetin-rutinoside isomer 2<br>13. apigenin-6,8-di-C-β-D-glucopyranoside/<br>luteolin-7-O-β-D-rutinoside<br>14. luteolin-7-β-D-glucuronide	[55]

	15. kaempferol 3-O-glucoside<br>16. patuletin 7-glucoside 17. dicaffeoylquinic acid isomer 1<br>18. dicaffeoylquinic acid isomer 2<br>19. dicaffeoylquinic acid isomer 3<br>20. apigenin-7-O-β-D-rutinoside<br>21. 4-succinyl-3,5-dicaffeoylquinic acid<br>22. kaempferol-7-O-rhamnoside<br>23. dicaffeoylquinic acid isomer 4<br>24. chrysoeriol 7-O-β-D-glucoside<br>25. kaempferol 3-O-β-D-glucopyranoside 6″-(3-hydroxy-3-methylglutarate)<br>26. acacetin 7-O-rhamnosylgalacturonide<br>27. 3,4-dihydroxyphenylacetic acid methyl ester<br>28. kaempherol/luteolin<br>29. quercetin 3′-methyl ether/6-methoxyluteolin<br>30. quercetin 3′-methyl ether/6-methoxyluteolin<br>31. tetrahydroxy-dimethoxyflavone<br>32. apigenin<br>33. luteolin methyl ether isomer 1<br>34. luteolin methyl ether isomer 2<br>35. 5,6,4′-Trihydroxy-7,3′-dimethoxyflavone/thymusin<br>36. 5,6,4′-Trihydroxy-7,3′-dimethoxyflavone/thymusin<br>37. trihydroxy-trimethoxyflavone isomer 1<br>38. trihydroxy-trimethoxyflavone isomer 2<br>39. scutellarein dimethyl ether isomer 1<br>40. dihydroxy-trimethoxyflavone<br>41. 8-methoxycirsilineol<br>42. scutellarein dimethyl ether isomer 2	
10 *Salvia* ssp.<br>Identified for the first time in *Salvia* spp. (in different species / with different extraction solvents).	1. ethyl gallate<br>2. 3′,4′,5′,7′-tetraxy droxy-3-methoxyflavone<br>3. hyperoside<br>4. isorhamnetin-glucoside<br>5. quercetin 3-glucuronide<br>6. rhamnetin<br>7. diosmetin	[3]
